# The non-muscle ADF/cofilin-1 controls sarcomeric actin filament integrity and force production in striated muscle laminopathies

**DOI:** 10.1016/j.celrep.2021.109601

**Published:** 2021-08-24

**Authors:** Nicolas Vignier, Maria Chatzifrangkeskou, Luca Pinton, Hugo Wioland, Thibaut Marais, Mégane Lemaitre, Caroline Le Dour, Cécile Peccate, Déborah Cardoso, Alain Schmitt, Wei Wu, Maria-Grazia Biferi, Naïra Naouar, Coline Macquart, Maud Beuvin, Valérie Decostre, Gisèle Bonne, Guillaume Romet-Lemonne, Howard J. Worman, Francesco Saverio Tedesco, Antoine Jégou, Antoine Muchir

**Affiliations:** 1 Sorbonne Université, INSERM, Institut de Myologie, Centre de Recherche en Myologie, 75013 Paris, France; 2 Department of Cell and Developmental Biology, University College London, London, UK; 3 Randall Centre for Cell and Molecular Biophysics, King’s College London, London, UK; 4 Université de Paris, CNRS, Institut Jacques Monod, 75013 Paris, France; 5 Sorbonne Université, UMS28, Phénotypage du Petit Animal, Paris, France; 6 Université de Paris, INSERM, CNRS, Institut Cochin, 75005 Paris, France; 7 Department of Medicine, Vagelos College of Physicians and Surgeons, Columbia University, New York, NY, USA; 8 Department of Pathology and Cell Biology, Vagelos College of Physicians and Surgeons, Columbia University, New York, NY, USA; 9 Dubowitz Neuromuscular Centre, UCL Great Ormond Street Institute of Child Health and Great Ormond Street Hospital for Children, London, UK; 10 The Francis Crick Institute, London, UK

**Keywords:** cofilin-1, ERK1/2 signaling, muscular dystrophy, skeletal muscle, sarcomeric organization

## Abstract

Cofilins are important for the regulation of the actin cytoskeleton, sarcomere organization, and force production. The role of cofilin-1, the non-muscle-specific isoform, in muscle function remains unclear. Mutations in *LMNA* encoding A-type lamins, intermediate filament proteins of the nuclear envelope, cause autosomal Emery-Dreifuss muscular dystrophy (EDMD). Here, we report increased cofilin-1 expression in *LMNA* mutant muscle cells caused by the inability of proteasome degradation, suggesting a protective role by ERK1/2. It is known that phosphorylated ERK1/2 directly binds to and catalyzes phosphorylation of the actin-depolymerizing factor cofilin-1 on Thr25. *In vivo* ectopic expression of cofilin-1, as well as its phosphorylated form on Thr25, impairs sarcomere structure and force generation. These findings present a mechanism that provides insight into the molecular pathogenesis of muscular dystrophies caused by *LMNA* mutations.

## Highlights

• Phosphorylation of cofilin-1 on Thr25 by p-ERK1/2 protects it from degradation by the UPS
• Phospho(T25)-cofilin-1 localizes on I-bands in sarcomeres
• Phosphorylation of cofilin-1 on Thr25 alters sarcomeric organization
• Phospho(T25)-cofilin-1 alter skeletal muscle force and lead to muscular dystrophy

## Graphical abstract

**Figure:**
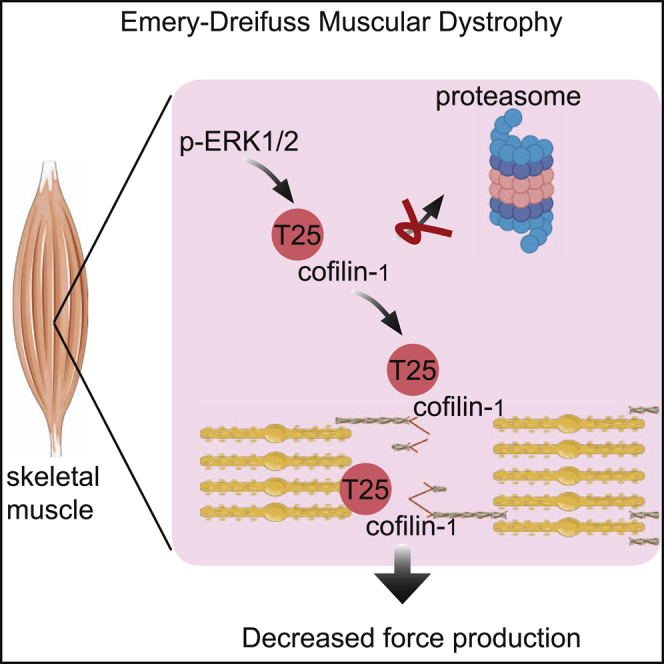

## Teaser

Extracellular signal-regulated kinase (ERK) 1/2 has been shown to be important for the development of muscular dystrophy. Vignier et al. find that active ERK1/2 catalyzes phosphorylation of cofilin-1 on Thr25 and protects it from proteasomal degradation, which physically disrupts sarcomeric organization and causes loss of muscle force generation.

## Introduction

*LMNA* encodes lamin A and lamin C, two components of the nuclear lamina that are essential for nuclear architecture and regulation of chromatin organization (Aebi et al., 1986; Dechat et al., 2008; Lin and Worman, 1993). *LMNA* mutations are responsible for autosomal forms of Emery-Dreifuss muscular dystrophy (EDMD) (Bonne et al., 1999), a disorder characterized by progressive muscle weakness and wasting associated with early contractures and dilated cardiomyopathy (Emery, 1987). *LMNA* mutations also cause limb girdle muscular dystrophy (Muchir et al., 2000), congenital muscular dystrophy (Quijano-Roy et al., 2008), or isolated cardiomyopathy without skeletal muscle involvement (Fatkin et al., 1999), expanding the phenotypic spectrum of striated muscle diseases linked to mutant A-type nuclear lamins.

Each skeletal muscle cell is composed of a repeated array of sarcomeres, the fundamental contractile units. Skeletal muscle cells are highly organized cells and necessary for voluntary movement induced by the somatic nervous system and to maintain posture. The coordinated contraction of all sarcomeres shortens the entire muscle cell and produces mechanical force. Despite the recent advances in deciphering the clinical description of muscular dystrophy caused by *LMNA* mutations (Madej-Pilarczyk, 2018), the molecular mechanisms leading to skeletal muscle damage remain to be determined. Loss of structural function and altered activation of tissue-specific signaling pathways have been proposed to partially explain the striated muscle dysfunction in these diseases (Brull et al., 2018). We have previously shown that the extracellular signal-regulated kinase 1/2 (ERK1/2) activity was altered in the affected skeletal muscles expressing disease-causing A-type lamins variants, thus participating in pathogenesis (Muchir et al., 2013). However, insights into the mechanisms bridging abnormal ERK1/2 activation and defective skeletal muscle function are lacking.

The actin cytoskeleton contributes to the functional and structural organization of cells. Actin filaments are also a main component of sarcomeres, which play an active role in the contractile force of muscle. The ADF/cofilin family comprises small actin-binding proteins with key roles in tissue homeostasis and disease. In mammals, three isoforms of the cofilin family have been described: ADF, cofilin-1, and cofilin-2 (Maciver and Hussey, 2002). The functions of each ADF/cofilin are not clearly defined, mostly because many of them may overlap. Cofilin-1 is a protein known to enhance actin filament turnover by severing and promoting dissociation of filamentous actin (F-actin) polymers into globular actin (G-actin) monomers (Bamburg and Bernstein, 2008). However, cofilin-2 was the only isoform reported to control the actin filaments turnover in mature muscle sarcomeres (Kremneva et al., 2014; Vartiainen et al., 2002). Here, we have uncovered a function of ERK1/2 signaling that catalyzes the phosphorylation of the cofilin-1 on Thr25, to protect it from degradation by the ubiquitination-proteasome pathway. This tight regulation of phospho(T25)-cofilin-1 protein levels by ERK1/2 signaling is important for the maintenance of sarcomere structure and force generation, which participate in the development of muscular dystrophy caused by *LMNA* mutations.

## Results

### Skeletal muscle pathology in soleus from *Lmna*^p.H222P/H222P^ mice

**Figure 1:**
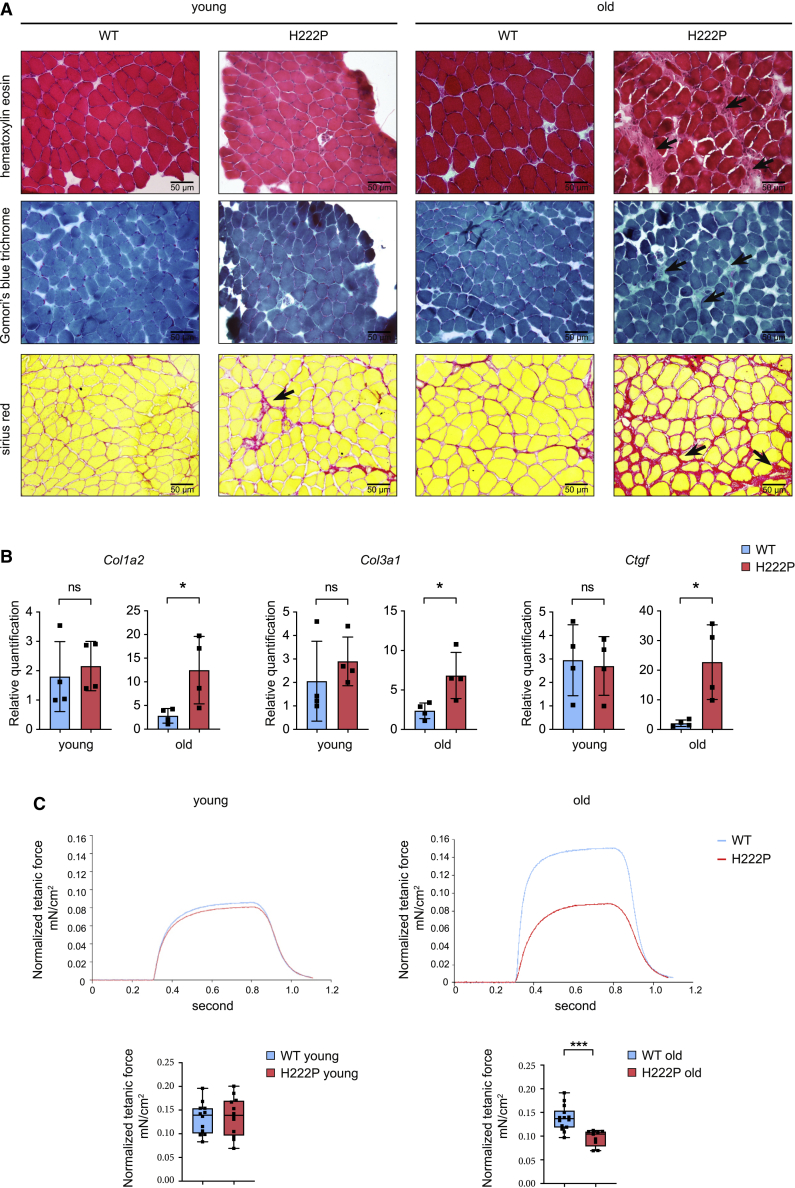
Abnormal soleus muscle structure and function in *Lmna*^p.H222P/H222P^ mice (A) Histochemical analysis of soleus from young and old wild-type (WT) and *Lmna*^p.H222P/H222P^ (H222P) mice. Sections of soleus muscles were stained with hematoxylin and eosin, modified Gomori’s trichrome, and Sirius red, showing an increase of fibrosis (arrows) in the old *Lmna*^p.H222P/H222P^ (H222P) mice. Scale bar, 50 μm. (B) Expression of fibrosis-related genes (*Col1a2*, *Col3a1*, and *Ctgf*) in the soleus from young and old WT (n = 4) and *Lmna*^p.H222P/H222P^ (H222P) (n = 4) mice. ^∗^p ≤ 0.01. Data are represented as mean ± SD. (C) Top: representative curves of tetanic forces of soleus from young and old, WT and *Lmna*^p.H222P/H222P^ (H222P) mice. Bottom: box-and-whisker plots showing median of tetanic forces of soleus from young, WT (n = 12) and *Lmna*^p.H222P/H222P^ (H222P) (n = 12) mice and old, WT (n = 14) and *Lmna*^p.H222P/H222P^ (H222P) (n = 9) mice. ^∗^p ≤ 0.01, ^∗∗∗^p ≤ 0.0001 between old WT and *Lmna*^p.H222P/H222P^ (H222P). Data are represented as mean ± SD.

Severe cytoarchitectural abnormalities of skeletal muscles, particularly in soleus muscle, associated with increased connective tissue have been previously observed in *Lmna*^p.H222P/H222P^ mice, a mouse model of muscular dystrophy caused by *LMNA* mutations (Arimura et al., 2005). We confirmed these findings by histological analyses in soleus muscles from young and old *Lmna*^p.H222P/H222P^ mice (Figure 1A). We also showed that the transcript levels of *Col1a2* and *Col3a1* encoding type I and III collagens, and *Ctgf* encoding connective tissue growth factor, were all correlated with the presence of interstitial fibrosis in the soleus muscle from old *Lmna*^p.H222P/H222P^ mice (Figure 1B). We next determined whether these changes in structural organization of soleus muscle could impede force generation. In conjunction with the dystrophic pattern, maximal force production was reduced in soleus muscle from old *Lmna*^p.H222P/H222P^ mice compared with wild-type (WT) mice (Table S1; Figure 1C). These structural and functional abnormalities were not observed in the fast twitch extensor digitorum longus (EDL) muscle from old *Lmna*^p.H222P/H222P^ mice (Figures S1A and S1B).

### Altered skeletal muscle actin dynamics in EDMD

To identify the molecular mechanisms that underlie skeletal muscle alterations, we performed transcriptomic analysis on soleus muscle along the course of the muscular dystrophy in *Lmna*^p.H222P/H222P^ mice. A principal-component analysis (Figure S2A) and a heatmap of unsupervised hierarchical cluster analysis (Figure S2B) performed on all the probes sets showed clear separation between young and old *Lmna*^p.H222P/H222P^ mice and WT mice. We next used a supervised learning method to distinguish probe sets representing genes with significant differences in expression. This analysis identified up and downregulated genes between young and old *Lmna*^p.H222P/H222P^ mice and WT mice and also along the progression of the disease for *Lmna*^p.H222P/H222P^ mice (Figures S2C and S2D; Table S2). These results were validated by reverse transcription quantitative polymerase chain reaction (RT-qPCR) (Figure S2E). We then analyzed functional class scoring, which improves sensitivity by statistically evaluating genes in biologically meaningful groups. Alteration of transcripts encoding genes relating to sarcomeric structure and structural organization of the muscle fiber was strongly correlated with the progression of the muscular dystrophy in *Lmna*^p.H222P/H222P^ mice (Figure S2F).

**Figure 2:**
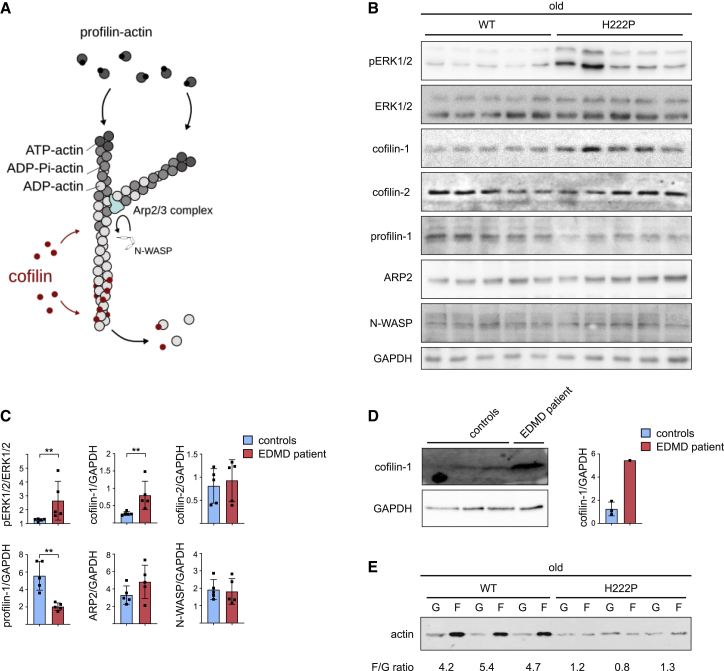
Increased cofilin-1 expression alters actin dynamics in Emery-Dreifuss muscular dystrophy (EDMD) (A) Schematic representation of actin dynamics mechanisms. (B) Immunoblots showing pERK1/2, ERK1/2, cofilin-1, profilin-1, ARP2, and N-WASP protein level in soleus from old WT (n = 5) and *Lmna*^p.H222P/H222P^ (H222P) (n = 5) mice. GAPDH is shown as loading control. (C) Quantification of pERK1/2, ERK1/2, cofilin-1, profilin-1, ARP2, and N-WASP protein expression level in soleus from old WT (n = 5) and *Lmna*^p.H222P/H222P^ (H222P) (n = 5) mice. ^∗∗^p ≤ 0.001 between old WT and *Lmna*^p.H222P/H222P^ (H222P). Data are represented as mean ± SD. (D) Immunoblots showing cofilin-1protein level in skeletal muscle from EDMD patient carrying *LMNA* mutation. GAPDH is shown as loading control. Data are represented as mean ± SD. (E) Immunoblot showing G-actin and F-actin protein levels in soleus from old WT (n = 3) and *Lmna*^p.H222P/H222P^ (H222P) (n = 3) mice.

We and others recently reported defective actin dynamics in cardiac muscle of mouse models of EDMD (Antoku et al., 2019; Chatzifrangkeskou et al., 2018; Ho et al., 2013). Therefore, we analyzed the expression of cofilin-1, cofilin-2, Neural Wiskott-Aldrich syndrome protein (N-WASP), actin-related protein 2 (ARP2), and profilin-1, proteins involved in the regulation of actin dynamics in soleus muscle (Figure 2A). We found increased cofilin-1 as well as decreased profilin-1 expression in the soleus muscle of old *Lmna*^p.H222P/H222P^ mice (Figures 2B and 2C). The expression of these proteins was not significantly affected at younger age (Figures S3A and S3B). Importantly, we also observed an activation of cofilin-1 expression in skeletal muscle from a patient with EDMD carrying the p.E358K *LMNA* mutation (Figure 2D). When examined by immunoblotting, the ratio of F-actin to G-actin was significantly lower in soleus muscle from old *Lmna*^p.H222P/H222P^ mice compared with WT mice (Figure 2E). These data indicate an actin filament disassembly in autosomal EDMD.

### Activated ERK1/2 signaling affects the stability of cofilin-1

**Figure 3:**
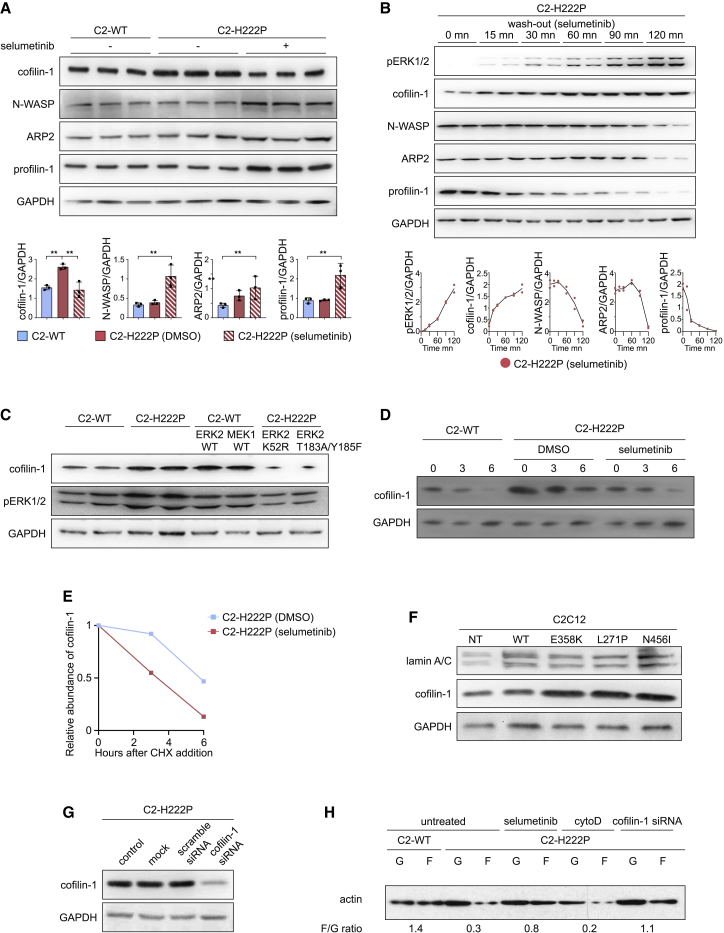
Increased cofilin-1 expression is under the control of ERK1/2 signaling (A) Representative immunoblots and quantification of cofilin-1, N-WASP, ARP2, and profilin-1 protein expression in C2C12 cells stably expressing WT (C2-WT) (n = 3) or p.H222P (C2-H222P) (n = 3) lamin A. GAPDH is shown as loading control. C2-H222P cells were either untreated or treated with selumetinib. ^∗∗^p ≤ 0.001 between C2-WT and C2-H222P ± selumetinib. Data are represented as mean ± SD. (B) Representative immunoblot and quantification of effects of washout of selumetinib on cofilin-1, N-WASP, ARP2 and profilin-1 protein expression level in C2-H222P cells. Data are represented as mean ± SD. (C) Representative immunoblot showing effects of transfection with ERK2 and MEK1 constructs on cofilin-1 protein expression in C2-WT and C2-H222P cells. GAPDH is shown as loading control. (D) Cycloheximide chase experiment using C2C12 cells stably expressing WT (C2-WT) or p.H222P (C2-H222P) lamin A, treated or not with selumetinib. Cells were treated with 50 μM cycloheximide and lysed at the indicated times for western blot analysis using anti-cofilin-1 antibody. GAPDH was used as a loading control. (E) Quantification of cofilin-1 signal intensity normalized to GAPDH content and expressed as the percent change from time zero, which was set at 100%. Data are represented as mean ± SD. (F) Representative immunoblots showing effects of transfection with different mutated lamin A constructs on cofilin-1 expression in C2C12 cells. (G) Representative immunoblot showing the effect of cofilin-1 siRNA on cofilin-1 expression. GAPDH is shown as a loading control. (H) Representative immunoblot showing the effect of cofilin-1 siRNA on G-actin and F-actin expression in C2-H222P cells. Cytochalasin D (cytoD) induces actin depolymerization.

These results raised the question whether the abnormal skeletal muscle activation of ERK1/2 signaling in muscular dystrophy caused by *LMNA* mutations was responsible for the increased cofilin-1 expression. We used stably transfected C2C12 mouse myoblasts expressing either WT (C2-WT) or the p.H222P lamin A variant (C2-H222P) (Choi et al., 2012), a simple model system where activation of ERK1/2 signaling has been well characterized (Chatzifrangkeskou et al., 2018; Choi et al., 2012; Muchir et al., 2013). We first investigated the levels of proteins involved in actin dynamics. We showed that cofilin-1 expression was increased in C2-H222P myoblasts compared with C2-WT cells (Figure 3A). Pharmacological inhibition of the ERK1/2 cascade with selumetinib, a selective MEK1/2 inhibitor, decreased cofilin-1 expression and increased N-WASP, ARP2, and profilin-1 levels (Figure 3A). Selumetinib washout experiments on C2-H222P cells suggested that regulation of cofilin-1, N-WASP, ARP2, and profilin-1 expression was related to ERK1/2 activity (Figure 3B). To further validate whether ERK1/2 regulated directly the protein levels of cofilin-1, we transiently transfected C2-WT cells with WT ERK2 or MEK1 constructs. This led to increased cofilin-1 protein levels compared with non-transfected cells (Figure 3C). Conversely, the inhibition of endogenous ERK2 in C2-H222P cells, upon transfection of kinase-dead (ERK2-K52R) or dominant-negative ERK2 (ERK2-T183A/Y185F) mutants constructs, led to decreased cofilin-1 expression compared with non-transfected C2-H222P cells (Figure 3C). Next, we analyzed the effect of ERK1/2 activity on cofilin-1 protein stability in a cycloheximide chase assay (Figures 3D and 3E). Cofilin-1 levels decreased at 6 h after the cycloheximide-mediated inhibition of protein synthesis in C2-H222P cells, indicating that cofilin-1 becomes unstable. We further showed that cofilin-1 protein levels decreased faster after the cycloheximide-mediated inhibition of protein synthesis in C2-H222P cells when the cells were treated with selumetinib. These results suggest that ERK1/2 signaling enhances cofilin-1 protein stability. We then assessed the role of ERK1/2 signaling on cofilin-1 expression *in vivo* using *Lmna*^p.H222P/H222P^ mice lacking Erk1 (Wu et al., 2014). We showed that the soleus muscles from these mice have a reduction of interstitial fibrosis compared with *Lmna*^p.H222P/H222P^ mice (Figure S4A and S4B). The inhibition of ERK1/2 cascade in *Lmna*^p.H222P/H222P^ mice lacking Erk1 led to a decreased cofilin-1 expression (Figures S4C and S4D). These data suggest that phosphorylation of ERK1/2 modulates cofilin-1 protein level. To identify whether other *LMNA* mutations have the same effect on cofilin-1 expression through pERK1/2 activation, we transiently transfected C2C12 cells with plasmids encoding lamin A variants found in EDMD (i.e., E358K, L271P, and N456I), which caused pERK1/2 activation (Figure 3F). The expression of these lamin A variants led to an elevated cofilin-1 protein level. These results suggest that *LMNA* mutations lead to increased cofilin-1 as a result of ERK1/2 hyperactivation. We next showed that depleting cofilin-1 by small interfering RNA (siRNA) in C2-H222P cells (Figure 3G) rescued the actin dynamics, as evidenced by normalization of the F/G-actin ratio compared with control (Figure 3H). This result demonstrates that the increased of cofilin-1 protein level in cells expressing disease-causing lamin A variants is responsible for actin depolymerization.

### Activated ERK1/2 signaling prevents cofilin-1 degradation through the proteasome

**Figure 4:**
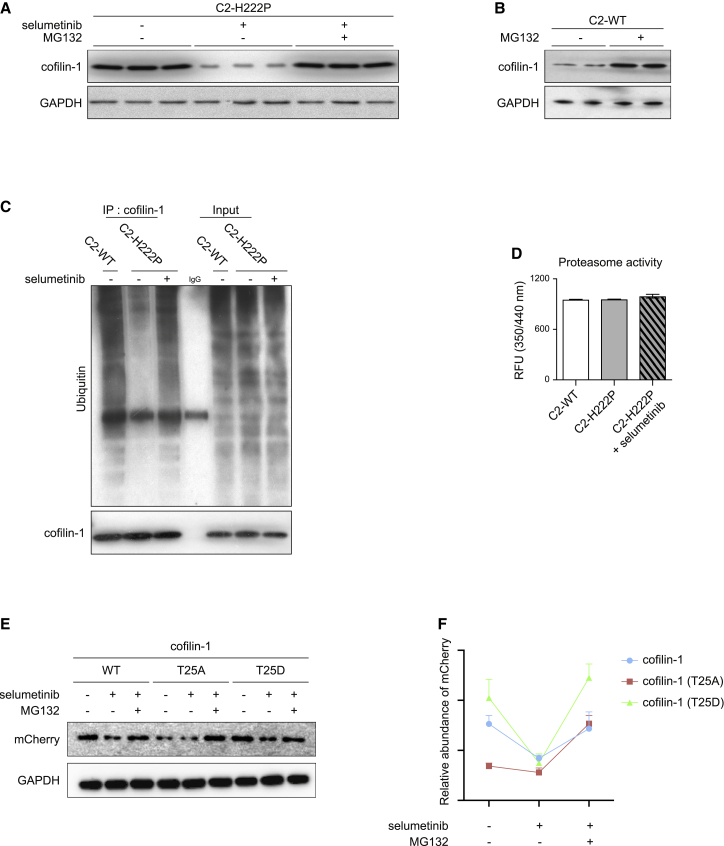
pERK1/2 protects cofilin-1 from degradation by the ubiquitin-proteasome pathway (A) Immunoblot showing effect of treatment with proteasome inhibitor MG132 on cofilin-1 expression in C2-H222P cells untreated or treated with selumetinib. GAPDH is shown as loading control. (B) Immunoblot showing effect of treatment with proteasome inhibitor MG132 treatment on cofilin-1 expression in C2-WT cells. GAPDH is shown as loading control. (C) Immunoprecipitation of cofilin-1 showing ubiquitination levels in C2-WT and C2-H222P cells untreated or treated with selumetinib. Input is shown as loading control. (D) Proteasome activity in C2-WT and C2-H222P cells untreated or treated with selumetinib. Data are represented as mean ± SD. (E) Immunoblot showing effect of selumetinib and MG132 on ectopically expressed mCherry-tagged cofilin-1, cofilin-1(T25A), and cofilin-1(T25D) in C2-WT cells. GAPDH is shown as loading control. (F) Quantification of mCherry signal intensity normalized to GAPDH content in C2-WT cells treated with the different conditions (n = 3). Data are represented as mean ± SD.

We next sought to identify the underlying mechanism that regulates cofilin-1 expression in cells expressing pathogenic lamin A variants. The ubiquitin proteasome pathway is responsible for the targeted degradation of proteins, such as cofilin-1 (Goldberg, 2003; Yoo et al., 2010). We thus speculated that pERK1/2 might protect cofilin-1 from proteasomal degradation. To test our hypothesis, we first examined the influence of the proteasome inhibitor MG132 on cofilin-1 expression. MG132 led to increased expression of endogenous cofilin-1 in C2-H222P cells compared with cells treated with selumetinib (Figure 4A). Similarly, treating C2-WT cells with MG132 increased endogenous cofilin-1 expression (Figure 4B). To further validate our hypothesis, we examined potential ubiquitination of cofilin-1 by immunoprecipitation. We showed a decreased ubiquitination of cofilin-1 in C2-H222P cells compared with C2-WT cells, which was prevented upon selumetinib treatment (Figure 4C). To rule out the possibility that this effect might arise from an impaired function of the proteasome in C2-H222P cells, we examined the proteasome activity in these cells. Protein extracts from C2-WT and C2-H222P cells showed no difference in proteasome activity and treatment of C2-H222P cells with selumetinib had no effect (Figure 4D). These data suggest that ERK1/2 signaling increases cofilin-1 stability by preventing its degradation via the proteasome-ubiquitin pathway. We recently showed that active phosphorylated ERK1/2 catalyzes the phosphorylation of the actin depolymerizing factor cofilin-1 on Thr25 (Chatzifrangkeskou et al., 2018). To test the stability of this phospho(T25)-cofilin-1, we ectopically expressed mCherry-tagged cofilin-1 as well as mCherry-tagged cofilin-1(T25A), a non-phosphorylatable variant, and cofilin-1(T25D), a phospho-mimetic variant, in C2-WT cells. While both cofilin-1 WT and cofilin-1(T25D) protein levels were decreased in cells treated with selumetinib compared with untreated cells, the expression of the mutant cofilin-1(T25A) remained unchanged (Figures 4E and 4F). Adding MG132 to the selumetinib treating cells rescued the expression of cofilin-1 variants (Figures 4E and 4F). These results strongly suggest that ERK1/2-mediated (T25)phophorylation protects cofilin-1 from proteosomal degradation. Given that other types of ERK1/2-dependent phosphorylation of cofilin-1 have been recently identified (Chatzifrangkeskou et al., 2018), it is possible that ERK1/2 signaling could regulate the activity of cofilin-1 through phosphorylation on other residues.

### Cofilin-1 controls sarcomere integrity

**Figure 5:**
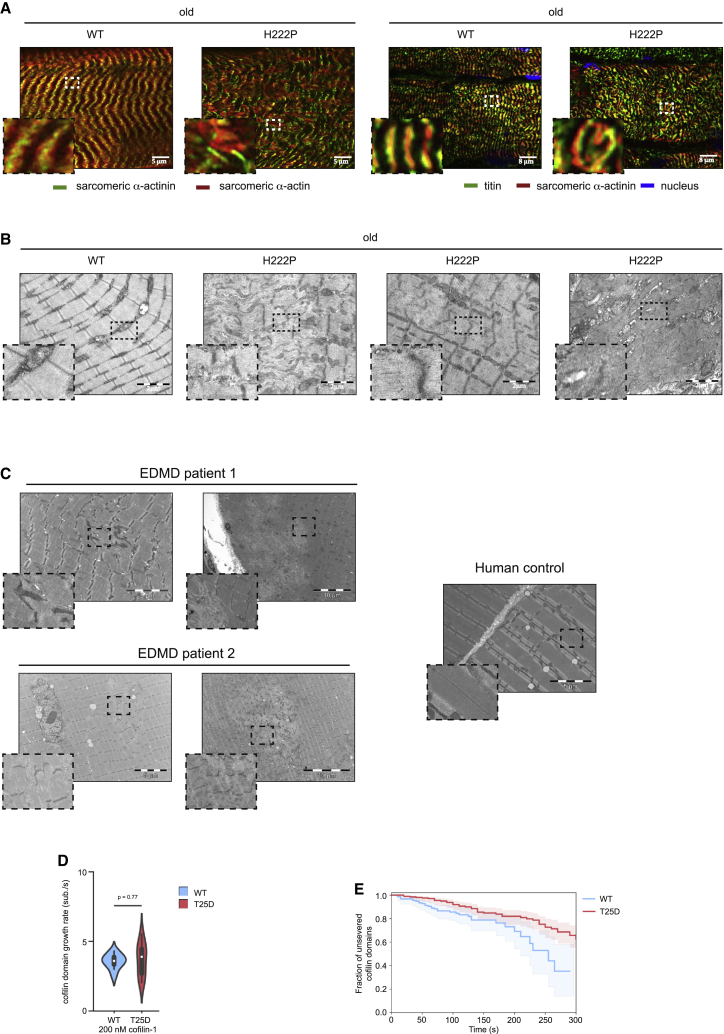
Alteration of sarcomere organization in EDMD (A) Left: immunofluorescence micrographs of sarcomeric α-actinin (green) and sarcomeric α-actin (red) labeled soleus muscle from old, WT and *Lmna*^p.H222P/H222P^ (H222P) mice. Scale bar, 5 μm. Right: immunofluorescence micrographs of titin (green) and sarcomeric α-actinin (red) labeled soleus muscle from old, WT and *Lmna*^p.H222P/H222P^ (H222P) mice. Scale bar, 8 μm. (B) Electron microscopy showing sarcomeric disorganization in soleus muscles from old WT and *Lmna*^p.H222P/H222P^ (H222P) mice. Scale bar, 2 μm. (C) Left: electron microscopy showing sarcomeric disorganization in striated muscles from EDMD patients carrying *LMNA* mutations. Right: striated muscle from human control. Scale bar, 10 μm (D) Growth rate of single cofilin domains for eGFP-cofilin-1 WT and T25D, observed on individual actin filaments *in vitro*, in the presence of 200 nM cofilin-1 (n = 10 filaments for each condition). The distributions are plotted as violin plots, with the white dot representing its median. The statistical test was a t test for the means of two independent samples with unequal variance. (E) Single eGFP-cofilin-1 WT or T25D domain severing rate. Time t = 0 is defined for every domain as the frame on which they nucleate. n = 152 and 202 cofilin domains for eGFP-cofilin-1 WT and T25D, respectively.

Given that members of the ADF/cofilin family have been reported to be essential regulators of actin dynamics in sarcomeres (Kremneva et al., 2014), we next assessed the sarcomere structure in skeletal muscles from EDMD. The structure of soleus muscle from old *Lmna*^p.H222P/H222P^ mice exhibited sarcomere disorganization compared with age-matched WT mice (Figures 5A and 5B). Sarcomere disorganization was not observed in soleus muscle from young *Lmna*^p.H222P/H222P^ mice (Figures S5A and S5B). Notably, similar sarcomere abnormalities were present in muscle biopsy specimens from EDMD patients carrying *LMNA* mutations (Figure 5C). This sarcomere disorganization was not observed in the fast-twitch EDL muscle from old *Lmna*^p.H222P/H222P^ mice (Figures S5C and S5D). We showed that these structural abnormalities were reduced in soleus muscle from *Lmna*^p.H222P/H222P^ mice lacking Erk1 (Figure S5E). To further validate this finding, we tested whether sarcomere abnormalities were also present in *LMNA* mutant human myotubes from striated muscle laminopathies. To this aim, we differentiated three human induced pluripotent stem cell (hiPSC) lines from patients with skeletal muscle laminopathies carrying *LMNA* p.K32del, p.L35P, and p.R249W mutations (Steele-Stallard et al., 2018) into skeletal myogenic cells (Maffioletti et al., 2015). Immunofluorescence analyses of sarcomeric proteins suggested that some abnormalities were detectable in the newly formed myotubes (Figure S6), albeit less evident than in the aforementioned EDMD muscle biopsy specimens (Figure 5C).

We then investigated whether the activation of cofilin-1 expression could contribute to myofilament disorganization. Transduction of WT differentiated primary mouse myofibers (Figure S7A) with an adeno-associated virus (AAV) encoding cofilin-1 increased the G-actin pool (Figure S7B) and led to altered myofibrillar organization compared with the untransfected condition (Figure S7C). We next hypothesized that phosphorylation of Thr25 on cofilin-1 has detrimental effects on striated muscle cells. Transduction with an adenovirus encoding cofilin-1(T25D) in WT differentiated primary mouse myofibers induced depolymerization of actin (Figure S7B) and altered myofibrillar organization (Figures S7C and S7D). However, transduction with an adeno-associated virus encoding cofilin-1(T25A) had no effect on both cellular actin dynamics (Figure S7B) and myofibrillar organization (Figures S7C and S7D). To study the role of cofilin-1 as well as its Thr25 phosphorylated form in muscle sarcomeres, we examined its localization in soleus muscle (Figures S8A and S8B). Immunofluorescence microscopy revealed regular striated pattern of cofilin-1 as well as its Thr25 phosphorylated form in WT mice (Figures S8A and S8B). In old *Lmna*^p.H222P/H222P^ mice, we observed a punctuated pattern alongside regular striated pattern (Figures S8A and S8B, arrows), reminiscent of sarcomere disorganization (Figures 5A and 5B). The co-localization with titin (antibody against PVEK domain) demonstrated that cofilin-1 as well as its Thr25 phosphorylated form localizes at I-bands in sarcomeres. These findings suggest that sarcomere disorganization arises from expression of phospho(T25)-cofilin-1, which participates in the development of muscular dystrophy caused by *LMNA* mutations.

To validate that cofilin-1 phosphorylated on Thr25 alters the sarcomeric organization, we assessed its molecular activity *in vitro* (Wioland et al., 2017), by exposing actin filaments to the phosphomimetic eGFP-cofilin1(T25D). We observed that cofilin-1(T25D) disassembles filaments, creating cofilin domains that grow similarly to WT (Figure 5D) although filaments severing at cofilin domain boundaries was slightly less efficient than WT cofilin-1 (Figure 5E). Overall, our *in vitro* results show that cofilin-1(T25D) is active and able to disassemble actin filament, as opposed to phosphomimetic cofilin-1(S3D) (Elam et al., 2017; Wioland et al., 2017). We thus conclude that phospho(T25)-cofilin-1 can alter actin dynamics and sarcomeric organization in striated muscle cells.

### Cofilin-1 is involved in muscle force generation *in vivo*

**Figure 6:**
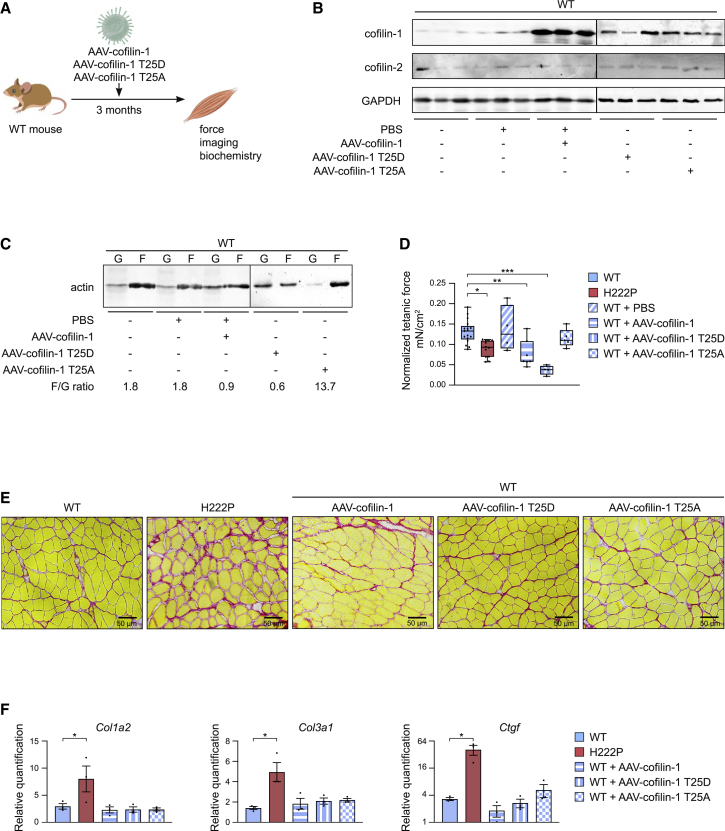
Cofilin-1 is involved in the muscle force generation *in vivo* (A) Schematic representation of the experimental procedure followed for transduction with AAV vectors expressing cofilin-1 constructs of soleus muscles in young WT mice. (B) Representative immunoblot of cofilin-1 protein levels in soleus from WT mice non-injected or injected with either PBS or AAV vector expressing cofilin-1 constructs. GAPDH is shown as loading control. (C) Representative immunoblot showing the effect of AAV expressing cofilin-1 construct on G-actin and F-actin expression in the soleus from WT mice non-injected or injected with either PBS or AAV vector expressing cofilin-1 constructs. (D) Tetanic force of soleus from WT (n = 17), *Lmna*^p.H222P/H222P^ (H222P) (n = 9) and WT mice injected with either PBS (n = 4) or AAV vectors expressing cofilin-1 (n = 7), cofilin-1(T25A) (n = 3) or cofilin-1(T25D) (n = 3). ^∗^p ≤ 0.01 between WT and *Lmna*^p.H222P/H222P^ (H222P), ^∗∗^p ≤ 0.001 between WT and WT AAV vectors expressing cofilin-1, ^∗∗∗^p ≤ 0.0001 between WT and WT AAV vectors expressing cofilin-1(T25D). Data are represented as mean ± SD. (E) Sirius Red staining of cross sections of soleus muscles from WT mice non-injected or injected with either PBS or AAV vector expressing cofilin-1 constructs. Section of soleus muscle from *Lmna*^p.H222P/H222P^ (H222P) is shown as control. Scale bar, 50 μm. (F) Expression of fibrosis-related genes (*Col1a2*, *Col3a1*, and *Ctgf*) in the soleus from WT mice non-injected or injected with either PBS or AAV vector expressing cofilin-1 constructs. Quantification of soleus muscle from *Lmna*^p.H222P/H222P^ (H222P) is shown as control. ^∗^p ≤ 0.01 between WT and *Lmna*^p.H222P/H222P^ (H222P). Data are represented as mean ± SD.

Force in striated muscles is produced by actin thin filaments sliding past the myosin thick filaments, resulting in sarcomere contraction (Huxley and Hanson, 1954; Huxley and Niedergerke, 1958). Given that elevated expression of cofilin-1 phosphorylated on Thr25 severely impacts the sarcomere structure, we hypothesized that it may affect the force production in skeletal muscles. We injected AAV expressing cofilin-1 into the soleus of 3-month-old WT mice (Figure 6A). Three months after injection, the expression of cofilin-1, but not cofilin-2, was increased in the injected soleus (Figure 6B), muscle actin dynamics was altered (Figure 6C), and myofilaments were disorganized (Figure S9). Tetanic force was significantly decreased in soleus muscles from cofilin-1-injected WT mice compared with non-treated WT mice (Figure 6D). Similar observations were made when soleus muscles were injected with AAVs expressing cofilin-1(T25D), but not AAVs expressing cofilin-1(T25A) (Figures 6B–6D). Fibrosis in WT soleus transduced with AAVs expressing cofilin-1(T25A) and cofilin-1(T25D) was unchanged compared with non-injected soleus (Figures 6E and 6F), demonstrating that fibrosis is not the reason for the reduced force production. In conclusion, we propose that cofilin-1 phosphorylated on Thr25 can impair skeletal muscle contractile properties by modulating sarcomeric actin dynamics.

## Discussion

**Figure 7:**
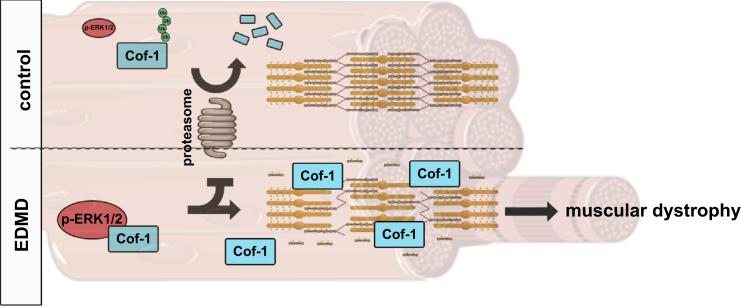
Schematic representation of the mechanism of pERK1/2-mediated protection of cofilin-1 from proteasome degradation and its consequences on sarcomeric actin depolymerization in striated muscle diseases caused by *LMNA* mutations

We have unraveled a protective role of phosphorylated ERK1/2 for cofilin-1 by blunting its degradation through the ubiquitination-proteasome pathway. This participates in the development of muscular dystrophy (Figure 7). Non-muscle cofilin-1 is a small actin-binding protein that accelerates actin turnover by disassembling actin filaments. In striated muscle cells, actin and several scaffolding and regulatory proteins are arranged into contractile myofilament (Ono, 2010). Actin-binding proteins are important for actin dynamics, which contribute to controlling myofilament structure and organization. This mechanism is regulated by mechanical forces (Fukuda et al., 2019; Skwarek-Maruszewska et al., 2009). The two ADF/cofilin isoforms, cofilin-1 and cofilin-2, are expressed in striated muscles, cofilin-2 being the dominant isoform (Ono et al., 1994; Vartiainen et al., 2002). We demonstrated that the non-muscle cofilin-1, as well as its Thr25 phosphorylated form, localizes on I-bands in sarcomeres and is an essential regulator of proper actin dynamics and sarcomeric organization. Abnormalities of actin dynamics hamper myofilament organization, alter skeletal muscle force, and ultimately lead to muscular dystrophy. In support of these findings, missense mutations in UNC-60B, the *C. elegans* homolog of ADF/cofilin, lead to defects in actin organization in the muscles (Ono et al., 1999).

Our work brings insight into a role played by actin regulators in muscular dystrophies. *LMOD3*, the gene encoding leimodin-3, a sarcomeric actin nucleator (Chereau et al., 2008), was identified as a cause of nemaline myopathies (Yuen et al., 2014). Similarly, mutated cofilin-2 has been previously shown to cause nemaline myopathy (Agrawal et al., 2012). Histopathological analysis of the skeletal muscle from cofilin-2 null mice revealed extensive myofilament disruptions (Agrawal et al., 2007). To date, cofilin-2 was the only known member of the ADF/cofilin family involved in the disassembly of actin skeletal muscle sarcomeres (Agrawal et al., 2012). Cofilin-1 and cofilin-2, both expressed in skeletal muscle cells, contribute to the dynamic turnover of F-actin (Kremneva et al., 2014). We recently showed that only cofilin-1 was phosphorylated on Thr25 by pERK1/2 and leads to disruption of myofilaments in the presence of *LMNA* mutations (Chatzifrangkeskou et al., 2018). Zebrafish cofilin-1 mutants also exhibit myofilament disruption associated with alteration of muscle contraction (Fukuda et al., 2019). Together, our results show that the ERK1/2-cofilin-1 axis regulates myofilament organization in skeletal muscles.

A higher level of ERK1/2 activity in skeletal muscle prevents the proteasome-dependent degradation of cofilin-1 protein, increasing its accumulation. Our results suggest that this escape from the proteasome machinery may be the consequence of increased phosphorylation of the cofilin-1 by ERK1/2 (Chatzifrangkeskou et al., 2018). Similarly, the stability of the FRA-1 is dependent on its phosphorylation status, which is regulated by ERK1/2 signaling (Casalino et al., 2003). In addition the proteasome-dependent degradation of c-FOS can be inhibited by ERK1/2 (Musti et al., 1997). Furthermore, loss of KLHL40 results in a severe lethal form of nemaline myopathy associated with destabilization of actin (Garg et al., 2014). KLHL40 binds to and stabilizes leimodin-3 by blocking its ubiquitination. In addition, loss of KLHL40 was associated with absence of leimodin-3 protein in skeletal muscle. It is now generally accepted that misregulation of the two major proteolytic systems, ubiquitin-proteasome and autophagy, can lead to abnormal protein accumulation that might be involved in the pathophysiology of several disorders, including muscular dystrophies (Sandri et al., 2013). In line with this, a previous report has suggested the involvement of autophagy in cardiomyopathy in autosomal EDMD (Choi et al., 2012). To develop therapeutic strategies for muscular dystrophy caused by *LMNA* mutations, it will be important both to identify and characterize mechanisms that regulate the proteasome-ubiquitination system and to find ways to prevent dysfunction of cell death mechanisms where abnormal protein aggregation has occurred. Altogether, these findings provide clues of a balance between protein stabilization and degradation, which are causing myopathies.

Our results indicate that cofilin-1-mediated alteration of actin dynamics is a cellular consequence of ERK1/2 activation in skeletal muscle cells. Specifically, we have shown that activation of ERK1/2 signaling protects cofilin-1 from proteasome-dependent degradation. We further demonstrated that muscular dystrophy is associated with muscle cofilin-1 activation, which generated sarcomere disruption and alteration of force production, suggesting that cofilin-1 overexpression is a pathological event in muscular dystrophy caused by *LMNA* mutations. Enhanced activation of cofilin-1 in skeletal muscles from a mouse model of EDMD and from patients with this disease supports our conclusion that this mechanism contributes to the pathology. Similar pathogenic mechanism of cofilin-1-mediated modulation of sarcomeric actin dynamics may play a role in other muscular dystrophies, in which there appears to be abnormal activation of ERK1/2 signaling (Barton, 2006; Griffin et al., 2005; Kumar et al., 2004, 2011; Lang et al., 2004; Muchir et al., 2007; Smythe and Forwood, 2012). These findings suggest that therapeutic approaches that could correct impaired actin dynamics may ameliorate muscular dystrophy caused by *LMNA* mutations.

## STAR★Methods

### Key resources table

**Table:** 

REAGENT or RESOURCE	SOURCE	IDENTIFIER
**Antibodies**		

Rabbit polyclonal to ERK1 + ERK2	abcam	Cat# 17942; RRID:AB_2297336
Mouse monoclonal Phospho-p44/42 MAPK (Erk1/2) (Thr202/Tyr204) (E10)	Cell Signaling	Cat# 9106; RRID:AB_331768
Rabbit monoclonal Cofilin (D3F9) XP®	Cell Signaling	Cat #5175; RRID:AB_10622000
Rabbit polyclonal Cofilin2	Invitrogen	Cat# PA5-22301; RRID:AB_11156612
Rabbit polyclonal Profilin1	Cell Signaling	Cat# 3237; RRID:AB_2236990
Rabbit polyclonal N-WASP (30D10)	Cell Signaling	Cat #4848; RRID:AB_10694415
Rabbit polyclonal ARP2	abcam	Cat# 47654; RRID:AB_1139848
Rabbit polyclonal Ubiquitin	Cell signaling	Cat# 3933; RRID:AB_2180538
Mouse monoclonal Actin (α-Sarcomeric)	Merck	Cat# A2172; RRID:AB_476695
Rabbit polyclonal Sarcomeric Alpha Actinin	abcam	Cat# 137346
Rabbit polyclonal pan actin	Cytoskelton	Cat# AAN01; RRID:AB_10708070
Mouse monoclonal Titin	DSHB	Cat# 9D10; RRID:AB_528491
Mouse monoclonal Lamin A/C (E-1)	Santa Cruz	Cat# sc-376248; RRID:AB_10991536
Mouse monoclonal Lamin A/C	Leica Biosystems	Cat# NCL-LAM-A/C; RRID:AB_563846
Mouse monoclonal GAPDH (6C5)	Abcam	Cat# 8245; RRID:AB_2107448
Goat Anti-Mouse IgG StarBright Blue 520	BioRad	Cat# 12005867
Goat Anti-Mouse IgG StarBright Blue 700	BioRad	Cat# 12004159; RRID:AB_2884948
Goat Anti-Rabbit IgG StarBright Blue 520	BioRad	Cat# 12005870; RRID:AB_2884949
Goat Anti-Rabbit IgG StarBright Blue 700	BioRad	Cat# 12004161; RRID:AB_2721073
Goat anti-Rabbit IgG (H+L) Cross-Adsorbed Secondary Antibody, Alexa Fluor 488	Invitrogen	Cat# A-11008; RRID:AB_143165
Goat anti-Rabbit IgG (H+L) Cross-Adsorbed Secondary Antibody, Alexa Fluor 546	Invitrogen	Cat# A-11010; RRID:AB_2534077
Goat anti-Rabbit IgG (H+L) Highly Cross-Adsorbed Secondary Antibody, Alexa Fluor Plus 405	Invitrogen	Cat# A48254; RRID:AB_2890548
Goat anti-Mouse IgG (H+L) Cross-Adsorbed Secondary Antibody, Alexa Fluor 488	Invitrogen	Cat# A-11001; RRID:AB_2534069
Goat anti-Mouse IgG (H+L) Highly Cross-Adsorbed Secondary Antibody, Alexa Fluor 546	Invitrogen	Cat# A-11030; RRID:AB_144695
Goat anti-Mouse IgG (H+L) Cross-Adsorbed Secondary Antibody, Alexa Fluor 405	Invitrogen	Cat# A-31553; RRID:AB_221604
Goat anti-Mouse IgM (Heavy chain) Cross-Adsorbed Secondary Antibody, Alexa Fluor 488	Invitrogen	Cat# A-21042; RRID:AB_141357
Goat anti-Mouse IgM (Heavy chain) Cross-Adsorbed Secondary Antibody, Alexa Fluor 546	Invitrogen	Cat# A-21045; RRID:AB_2535714

**Bacterial and virus strains**		

DH5α Competent Cells	ThermoFisher Scientific	EC0112

**Biological samples**		

Human skeletal muscle tissue	Kindly provided by Myobank-AFM, Paris, France.	23-year-old patient carrying the *LMNA* p.R249Q mutation
Human skeletal muscle tissue	Kindly provided by Myobank-AFM, Paris, France.	14-year-old patient carrying the *LMNA* p.E358K mutation
Human skeletal muscle tissue	Kindly provided by Myobank-AFM, Paris, France.	Age match controls
129S2/svPasCrl mouse primary myoblastes	This paper	N/A
129S2/svPasCrl *Lmna*^p.H222P/H222P^ mouse primary myoblastes	This paper	N/A
Rabbit muscle acetone powder	Pel-Freeze	41995 −1
Human erythrocytes	Etablissement Francais du Sang	N/A

**Chemicals, peptides, and recombinant proteins**		

Mouse eGFP-cofilin-1	Kremneva et al., 2014	Uniprot: P18760
Mouse eGFP-(T25D)-cofilin-1	This paper	N/A
Alexa488- succimidyl ester	Life Technologies	Cat#A20000

**Critical commercial assays**		

Proteasome activity assay kit	Abcam	ab107921
G-actin/F-actin *in vivo* assay kit	Cytoskeleton	BK037
RNeasy Mini Kit	QIAGEN	74104
Agilent RNA 6000 Nano Kit	Agient	5067-1511
SuperScript® III First-Strand Synthesis System for RT-PCR	Invitrogen	18080-051
LightCycler® 480 SYBR Green I Master	Roche	04887352001
GeneChip Mouse Gene 2.0 ST Array	ThermoFisher Scinetific	902119
GeneChip WT Pico Kit	ThermoFisher Scinetific	902622
QuikChange II Site-Directed Mutagenesis Kit	Agilent	200523
Lipofectamine 2000 Transfection Reagent	ThermoFisher Scinetific	11668019

**Deposited data**		

Transcriptome analysis	Affimetrix GeneChip Mouse Gene 2.0 ST Array	GEO number: GSE146112

**Experimental models: Cell lines**		

C2C12 immortalized mouse myoblast cell lines that constitutively expressed wild type *Lmna* gene	Kindly provided by Dr. Howard J. Worman, Department of Medicine, College of Physicians and Surgeons, Columbia University, New York, New York 10032, USA	N/A
C2C12 immortalized mouse myoblast cell lines that constitutively expressed mutated c.665A > C *Lmna* gene	Kindly provided by Dr. Howard J. Worman, Department of Medicine, College of Physicians and Surgeons, Columbia University, New York, New York 10032, USA	N/A
(hiPSC) lines from patients carrying *LMNA* p.K32del	Cellular Dynamics International Inc. and Cure Congenital Muscular Dystrophy (CureCMD; https://www.curecmd.org)	https://fujifilmcdi.com https://www.curecmd.org
(hiPSC) lines from patients carrying *LMNA* p.L35P	Cellular Dynamics International Inc. and Cure Congenital	https://fujifilmcdi.com https://www.curecmd.org
(hiPSC) lines from patients carrying *LMNA* p.R249W	Cellular Dynamics International Inc. and Cure Congenital	https://fujifilmcdi.com https://www.curecmd.org
C2C12 myoblast cell line	ATCC	CRL-1772
Human iPSCs: Healthy Control UCLi007-A	Tedesco laboratory (https://hpscreg.eu/cell-line/UCLi007-A)	UCLi007-A or STM2012CTRL03(401)

**Experimental models: Organisms/strains**		

Mouse 129S2/svPasCrl wild-type	Janvier Labs	https://www.janvier-labs.com
Mouse 129S2/svPasCrl *Lmna*^p.H222P/H222P^	Kindly provided by Dr. Gisèle Bonne, INSERM UMR-S 974, Paris, France	N/A
Mouse C57BL/6JRj wild type	Janvier Labs	https://www.janvier-labs.com
Mouse C57BL/6JRj *Lmna*^p.H222P/H222P^,*ERK1*^KO/KO^	Kindly provided by Dr. Howard J. Worman, Department of Medicine, College of Physicians and Surgeons, Columbia University, New York, New York 10032, USA	N/A

**Oligonucleotides**		

List of primers	This paper	Table S3
SignalSilence® Cofilin siRNA I	Cell Signaling	Cat #6267

**Recombinant DNA**		

virus: AAV-rh10-Cfl1	Kindly provided by Dr. Maria-Grazia Bieferi, INSERM UMR-S 974, Paris, France	N/A
virus: AAV-rh10-Cfl1(p.T25A)	Kindly provided by Dr. Maria-Grazia Bieferi, INSERM UMR-S 974, Paris, France	N/A
virus: AAV-rh10-Cfl1(p.T25D)	Kindly provided by Dr. Maria-Grazia Bieferi, INSERM UMR-S 974, Paris, France	N/A
plasmid: GFP-ERK2	Kindly provided by P. Stork, Oregon Health and Science University	N/A
plasmid: RFP-MEK1	Kindly provided by P. Stork, Oregon Health and Science University	N/A
plasmid: GFP-ERK2^K52R^	Kindly provided by P. Stork, Oregon Health and Science University	N/A
plasmid: GFP-ERK2^T183A/Y185F^	Kindly provided by P. Stork, Oregon Health and Science University	N/A
plasmid: GFP-lamin A	Ostlund et al., 2001	N/A
plasmid: GFP-lamin A^E358K^	Ostlund et al., 2001	N/A
plasmid: GFP-Lamin A^L271P^	Ostlund et al., 2001	N/A
plasmid: GFP-Lamin A^N456I^	Ostlund et al., 2001	N/A
plasmid: pmCherryC1-Cofilin1	Addgene	Cat #27687
plasmid: pmCherryC1-Cofilin1^T25A^	This paper	N/A
plasmid: pmCherryC1-Cofilin1^T25D^	This paper	N/A
plasmid: Mouse eGFP-cofilin-1	Kremneva et al., 2014	Uniprot: P18760
plasmid: Mouse eGFP-(T25D)cofilin-1	This paper	Uniprot: P18760

**Software and algorithms**		

ImageJ	Schneider et al., 2012	https://imagej.net/
Prism 8	GraphPad Software, LLC	https://www.graphpad.com
GeneChip Command Console Software	ThermoFisher Scientific	https://www.thermofisher.com/us/en/home.html
Transcriptome Analysis Console (TAC) Software	ThermoFisher Scientific	https://www.thermofisher.com/us/en/home.html
Linear Models for Microarray and RNA-Seq Data	Ritchie et al., 2015	https://bioconductor.org/packages/release/bioc/html/limma.html
Database for Annotation, Visualization and Integrated Discovery (DAVID)	Huang et al., 2009	https://david.ncifcrf.gov/
ErmineJ	Lee et al., 2005	https://erminej.msl.ubc.ca
GenEx software	multid	https://multid.se
PowerLab System 4SP	AD Instruments	https://www.adinstruments.com
Labchart 4 v8	AD Instruments	https://www.adinstruments.com
Jupyter for numpy/python analysis	Jupyter	https://jupyter.org
GeneTraffic 3.0	Stratagene	https://www.stratagene.com

### Resource availability

#### Lead contact

Further information and requests for resources and reagents should be directed to and will be fulfilled by the lead contact Antoine Muchir (a.muchir@institut-myologie.org)

#### Materials availability

In this work, the newly generated material is listed in the Key resources table and can be shared upon request.

### Experimental model and subject details

#### Human skeletal muscles

Human skeletal muscles were obtained from the Myobank-AFM, Paris, France (https://www.institut-myologie.org/en/recherche-2/myobank-afm/). Patients and age-match controls were informed and gave consent. For biochemistry, we studied skeletal muscle from a 14-year-old man subject carrying the *LMNA* p.E358K mutation. For electron microscopy, we studied skeletal muscle from a 12-year-old man subject carrying the *LMNA* p.H222P mutation and a 23-year-old man carrying the *LMNA* p.R249Q mutation. Tissue samples without patient identifiers received from consent donors were not obtained specifically for this study.

#### Animals

All *in vivo* experiments were approved by the French Ministry of Agriculture (approval number #6455 and #20161). The animal experiments were performed according to the guidelines from Directive 2010/63/EU of the European Parliament on the protection of animals used for scientific purposes. Wild-type and Lmnap.H222P/H222P mice from the 129S2/SvPasCrl genetic background (Vignier et al., 2019) were housed in a disease-free barrier facility with 12 h/12 h light/dark cycles and received chow diet and water *ad libitum*. Young mice correspond to 2-4 months old animals, and old mice to 6-8 months old animal. Only males were chosen since the onset of the disease was earlier than in females (Arimura et al., 2005).

#### Animal skeletal muscles

Animal skeletal muscles, soleus and/or EDL were harvested from dead, young and old, wild-type, Lmnap.H222P/H222P mice and Lmnap.H222P/H222P mice lacking ERK1. Animals were sacrifice by cervical dissociation according to the guidelines from Directive 2010/63/EU of the European Parliament on the protection of animals used for scientific purposes.

#### Cell lines

C2C12 mouse myoblast cell line was purchased at ATCC (RRID:CVCL_0188). Cells were cultured in Dulbecco’s Modified Eagle’s Medium (DMEM) supplemented with 10% fetal bovine serum in 5% CO2 and 20% O2 at 37°C.

C2C12 mouse myoblast cell lines that constitutively expressed wild-type Lmna gene (C2-WT) and mutated c.665A > C Lmna gene (C2-H222P) has been described previously (Choi et al., 2012). C2-WT and mutated C2-H222P were kindly provided by Dr. Dr. Howard J. Worman, Department of Medicine, College of Physicians and Surgeons, Columbia University, New York, New York 10032, USA. Cells were cultured in Dulbecco’s Modified Eagle’s Medium (DMEM) supplemented with 10% fetal bovine serum in 5% CO2 and 20% O2 at 37°C. Cells were treated with 50 μM selumetinib (8 hours), 10 μM MG132 (5 hours), 10 μg/ml cycloheximide, 100 nM cytochalasin D, 10 μg/ml CHX for the indicated time points.

LMNA mutant human iPSCs were kindly provided by Cellular Dynamics International Inc. (CDI; https://cellulardynamics.com) and Cure Congenital Muscular Dystrophy (CureCMD; https://www.curecmd.org). Human iPSC-derived myogenic cells were cultured and differentiated according to previously reported protocol (Maffioletti et al., 2015). In brief, cells were plated onto Matrigel-coated dishes and upon achievement of 90%–100% confluence, differentiation was induced by pulsing cells twice with 1μM 4-OH tamoxifen: once in proliferation medium and the second time after 24 hours in differentiation medium (i.e., DMEM with 2% horse serum). Differentiation was maintained for 4 days changing the medium every other day. Work with human cells in the Tedesco laboratory was performed under approval of the NHS Health Research Authority Research Ethics Committee reference no. 13/LO/1826; IRAS project ID no. 141100.

#### Mouse primary cells

Mouse primary myoblast cells were obtained from 129S2/svPasCrl wild-type mouse. Primary mouse myoblasts were cultured on collagen-coated dishes in Ham’s F10 medium (Life Technologies) supplemented with 20% FBS (Eurobio), 2ng/ml basic fibroblast growth factor (FGF) (R&D Systems) and 1% penicillin/streptomycin (Pen/Strep) (Life Technologies). Myoblasts were plated at a density of ∼1 × 104 cells per cm2. After 48 h, myoblasts were shifted to 5% horse serum and left to differentiate.

### Method details

#### Adeno-associated virus (AAV) delivery

AAV delivery was performed by intramuscular injection using a 29-G needle, at the dose of 1.1x1011 viral particles per 5 mg of tissue in a final volume of 5 μl. Phosphate-buffered saline; PBS 1X (137 mM NaCl, 2.7 mM KCl, 10 mM Na2HPO4, 1.8 mM KH2PO4) was use as placebo. Mice were anesthetized with intraperitoneal injection of xylazin (10 mg/kg)/ketamine (100 mg/kg) cocktail and place on a heating pad at 28°C during the intervention. Wild-type males were injected with AAV-rh10-Cfl1, AAV-rh10-Cfl1(p.T25A) and AAV-rh10-Cfl1(p.T25D) at 90 days of age.

AAV vectors of serotype rh10 (AAV-rh10-Cfl1, AAV-rh10-Cfl1(p.T25A), AAV-rh10-Cfl1(p.T25D)), carrying sequence of the wild-type cofilin-1 gene (accession NM_007687), cofilin-1 c.73A>G or cofilin-1 c.73ACA>GAT under the control of the cytomegalovirus immediate/early promoter was prepared by the triple transfection method in HEK293T cells as previously described (Biferi et al., 2017; Chatzifrangkeskou et al., 2018).

#### RNA isolation and reverse-transcription qPCR

Total RNA was extracted using the RNeasy isolation kit (QIAGEN) according to the manufacturer’s instructions. Adequacy and integrity of extracted RNA were determined with the 2100 Bioanalyzer system (Agilent) according the manufacturer’s instructions. cDNA was synthesized using the SuperScript III first-strand synthesis system according to the manufacturer’s instructions (Invitrogen). Real-time qPCR reactions were performed with SYBR Green I Master mix (Roche) using the LightCycler® 480 (Roche). Relative levels of mRNA expression calculated using the ΔΔCT method were normalized to housekeeping mRNA (Pfaffl, 2001).

#### Microarray processing

Transcriptome analysis was performed with GeneChip Mouse Gene 2.0 ST Array (Affymetrix), which contains 698,000 probes that covered 35,240 transcripts from RefSeq database. Complementary DNA synthesis, cRNA synthesis, and labeling were performed with GeneChip WT Pico Reagent Kit (Applied Biosystems) according to the manufacture’s instructions. Hybridization, washing, staining and scanning of arrays were performed at the GeneChip Core Facility of the Cochin Hospital (GENOM’IC). Image files were obtained through Affymetrix GeneChip software and analyzed by robust multichip analysis using Affymetrix microarray “.cel” image files and GeneTraffic 3.0 software (Stratagene). Gene expression analysis was realized using the Affymetrix Transcriptome Analysis Console (TAC). Genes were processed and normalized using the Robust Multichip Analysis (RMA), which consists in three steps: background correction, normalization, and probe set summarization (Irizarry et al., 2003). Paired comparisons between groups were realized using Limma (Ritchie et al., 2015) and the generated p values were corrected with Benjamini and Hochberg procedure (Reiner et al., 2003). Genes were finally identified as being differentially expressed (DEG) if they met a false discovery rate threshold of p < 0.05 and showed at least a 2-fold difference in expression independent of absolute signal intensity. Gene expression changes related to functional groups were analyzed using the class score method in the bioinformatics tools DAVID (https://david.abcc.ncifcrf.gov/) and ErmineJ (http://www.chibi.ubc.ca/ermineJ/) to provide a statistical confidence to groupings. These bioinformatics tools take as input the q-values of differentially expressed genes and identify statistically significant functional groupings (GO terms) using modified Fisher exact test in DAVID and Wilcoxon rank-sum test in ErmineJ. Significant GO terms were identified using a false discovery rate of p < 0.05.

#### Plasmids

Plasmids encoding GFP-ERK2, RFP-MEK1, GFP-ERK2 K52R and GFP-ERK2 T183A/Y185F were kindly provided by P. Stork (Oregon Health and Science University). Plasmids encoding GFP-lamin A, GFP-lamin A E358K, GFP-Lamin A L271P and GFP-LaminA N456I have been previously described (Ostlund et al., 2001). Cofilin-pmCherryC1 was purchased from Addgene (#27687). Mutagenesis was carried out using QuikChange II Site-Directed Mutagenesis Kit (Agilent Technologies) as previously described (Chatzifrangkeskou et al., 2018).

Transient transfection experiments were performed using Lipofectamine 2000 (Invitrogen) according to the manufacturer’s instructions. Briefly, cells were seeded at 3 × 105 cells per well in 6-well plates or at 1 × 106 cells per 10 cm Petri dish were transfected with 3 μg or 15 μg plasmid DNA respectively for 24 h.

#### Cofilin-1 binding and severing activity

The procedure is described in detail in Wioland et al. (2017). Briefly, single, fluorescently labeled, rabbit alpha-skeletal actin filaments are aged for 15 minutes and exposed to eGFP-cofilin-1 in a microfluidics chamber, in F-buffer (5 mM Tris HCl pH 7.4, 50 mM KCl, 1 mM MgCl2, 0.2 mM EGTA, 0.2 mM ATP, 10 mM DTT and 1 mM DABCO), at room temperature (RT). Acquisition is performed on a Nikon TiE inverted microscope, controlled by micromanager, using epifluorescence with a 120W Xcite lamp (Lumen dynamics) and images were acquired by an sCMOS Orca-Flash4.0 camera (Hamamatsu). All experiments were performed at least twice and at least one representative movie was analyzed as described in (Wioland et al. (2017). The Welch’s unequal variances t test was used to test for significant differences in the domain growth rates, using the ‘scipy’ python package.

#### Protein extraction and immunoblotting

Total proteins from mouse soleus tissue or cultured cells were extracted in Cell lysis buffer (Cell Signaling) completed with protease inhibitors (25 mg/ml aprotinin; 10 mg/ml leupeptin; 1 mM 4-[2-aminoethyl]-benzene sulfonylfluoride hydrochloride; 2 mM Na3VO4). The lysates were sonicated (3 pulses of 10 s at 30% amplitude) and protein concentration was measured with the Bicinchoninic Acid Assay protein assay (Pierce). Protein extracts were separated by 10% sodium dodecyl sulfate–polyacrylamide gel electrophoresis (SDS-PAGE) and transferred onto nitrocellulose membranes (Invitrogen). Blocking and antibody incubations were performed in 5% bovine serum albumin. Membranes were incubated with fluorescent-conjugated anti-mouse or anti-rabbit secondary antibodies (BioRad) 1h at RT. Antibody detection was imaged using the ChemiDoc Touch Imaging System (BioRad). Quantification was done using ImageJ software.

#### F/G actin ratio measurements

The ratio of F-actin to G-actin was determined using the G-actin/ F-actin *in vivo* assay kit (Cytoskeleton) according to the manufacturer’s instructions. Briefly, 2 mg of protein from cells or frozen soleus tissues were homogenized in Lysis and F-actin Stabilization Buffer and centrifuged at 2,000 rpm for 5 min to remove unbroken cells. F-actin was separated from G-actin by centrifugation at 100,000 g for 60 min at 37□°C. The F-actin- containing pellet was resuspended in F-actin Depolymerizing Buffer at a volume equivalent to the G-actin-containing supernatant volume. The resuspended F-actin pellet was kept on ice for 60 min and was gently mixed every 15 min to dissociate F-actin. Proteins in equivalent volumes (10 μl) of supernatant and pellet were separated by SDS-PAGE and subjected to immunoblot analysis using an anti-pan actin antibody supplied in the kit. F/G actin ratio was quantified using ImageJ software.

#### Proteasome activity assay

Protein were extracted from C2-WT and C2-H222P cells in PBS 1X-0.5% NP-40. Proteasome activity was measured by proteasome activity assay kit (Abcam ab107921) according to the manufacturer’s instructions. Fluorescence was measured with a plate reader (BMG Labtech) in the presence/absence of MG132 at Ex/Em = 350/440 nm at 37°C.

#### Immunoprecipitation

Cells were treated with 10 μM MG132 and lysed in TBS 1X-1mM EDTA-1% NP-40 completed with proteinase inhibitor cocktail (Roche). Cell lysates were incubated with 20 μL protein A Dynabeads (Invitrogen) and 2 μg of the indicated antibodies for 2 h at 4°C. Pelleted beads were collected in sample buffer NuPAGE LDS (Thermo Fisher Scientific) with 200 mM DTT and subjected to SDS-PAGE and immunoblotting.

#### Histological analyses

The skeletal muscles were briefly washed in PBS and snap frozen at −70°C in isopentan. Frozen tissues were cut into 8-μm-thick sections. Haematoxylin and eosin, Gomori’s trichrome and Sirius Red stainings were performed according to standard procedures.

#### Immunofluorescence microscopy

Frozen tissues were cut into 8-μm-thick sections. Cryosections were fixed (PBS 1X, 4% PFA) 15 min at RT, permeabilized (PBS 1X, 0.5% Triton X-100) 10 min at RT and blocked (PBS 1X, 0.3% Triton X-100, 5% BSA) one hour at RT. Sections were incubated overnight at 4oC with primary antibodies (PBS 1X, 0.1% Triton X-100, 1% BSA) and washed in PBS 1X. Sections were then incubated for 1 h with secondary anti-rabbit or anti-mouse IgG conjugated with Alexa Fluor 488 or Alexa Fluor 546 antibodies. Sections were washed with PBS 1X and slides were mounted in Vectashield mounting medium with DAPI (Vector Laboratories). For human iPSC-derived myotubes, cells were fixed using 4% PFA at RT for 10 min. Samples were permeabilized (PBS 1X, 0.2% Triton X-100, 1% BSA) 45 min at RT and blocked (PBS 1X, 10% goat serum, 0.2% Triton X-100, 1% BSA) 30 min at RT. Samples were incubated overnight at 4°C with primary antibodies and washed three times (PBS 1X, 0.2% Triton X-100). Cells were incubated one hour at RT with secondary antibodies goat anti-mouse IgG, goat anti-mouse IgM, goat anti-rabbit IgG conjugated with Alexa Fluor 405, 488 and 546, respectively. Cells were washed with PBS 1X and slides were mounted in Vectashield mounting medium with DAPI (Vector Laboratories). Imaging of iPSC-derived myotubes was carried out using a Leica SPE inverted confocal microscope using a 63X lens and selecting random fields. Scoring of myotubes carrying sarcomere abnormalities and α-actinin or titin aggregates was performed blindly. The myofibers were considered disorganized when they showed no sarcomere organization/pattern across most of the myotube, lack of myofibrils-like structures and abundance of aggregates.

#### Electron microscopy

Freshly harvested skeletal muscles were cut into small pieces and immediately fixed (PBS 1X, 2.5% glutaraldehyde) 1 h at RT. After washing in PBS 1X, samples were post-fixed (PBS 1X, 1% OsO4), dehydrated in a graded series of acetone and embedded in an epoxy resin. Ultrathin sections were cut at 90 nm and stained with uranyl acetate and lead citrate, examined using a transmission electron microscope (JEOL 1011) and photographed with a digital Erlangshen 1000 camera (GATAN), using Digital Micrograph software.

#### Contractile properties of isolated muscle *in vitro*

The isometric contractile properties of soleus and EDL muscles were studied *in vitro* according to previously published protocols (Agbulut et al., 2009; Mouisel et al., 2014). Muscles were immersed in an oxygenated (95% O2 and 5% CO2) Tyrode solution (58.5 mM NaCl, 24 mM NaHCO3, 5.4 mM KCl, 1.2 mM KH2PO4, 1.8 mM CaCl2, 1 mM MgSO4, and 10 mM glucose (pH 7.4)) at 22°C. Muscles were connected at one end to an electromagnetic puller and at the other end to a lever arm of a servomotor system (Isometric Force Transducer FT50, Harvard Apparatus). The skeletal muscles were placed between two electrodes parallel to the muscle. Once the system was equilibrated (30 min), an electrical field was applied to the muscle. Twitch and tetanic contractions were performed and data were recorded and analyzed using the PowerLab System (4SP; AD Instruments) and software (Labchart 4 v8; AD Instruments). Absolute maximal isometric tetanic force (P0) was measured during tetanic contractions (frequency of 120 Hz, train of stimulation of 500 ms). The muscle length was adjusted to the optimum length (L0) that produced P0. Specific maximal isometric force (sP0) was calculated by dividing the force by the estimated cross-sectional area (CSA) of the muscle. Assuming muscles have a cylindrical shape and a density of 1.06 mg/mm3, the muscle cross-sectional area corresponds to the wet weight of the muscle divided by its fiber length (Lf). The fiber length to L0 ratio of 0.70 was used to calculate Lf (Mendias et al., 2006). Isometric twitch contractions were recorded at L0. The following parameters of the twitch contraction were measured: maximum twitch force (Pt), time to peak tension (TTP ms) and half relaxation time (HRT ms). Specific Pt (sPt mM/mm2) was calculated by dividing the force by the CSA of the muscle.

### Quantification and statistical analysis

Immunoblots and gels were analyzed and quantified with Fiji software. Statistical analyses were performed using GraphPad Prism software. Statistical significance between groups of mice was analyzed with a corrected non-parametric test, Mann-Whitney test when compared two sets of data, or Kruskal Wallis test with Dunn’s test post-test when compared multiple sets data. The statistical details of experiments are presented in the relevant figure legends. A *p* value of < 0.05 was considered significant.

## Data Availability

• Affymetrix array transcriptome data are available at the GEO database: GSE146112 (https://www.ncbi.nlm.nih.gov/geo/query/acc.cgi?acc=GSE146112**)** • This paper does not report original code • Any additional information required to reanalyze the data reported in this paper is available from the lead contact upon request.

## Supplemental information

Document S1. Figures S1–S9 and Table S1

Table S2. Genes with altered expression as defined by p value < 0.01 and −2 ≤ fold change ≥ 2 in soleus between young and old Lmnap.H222P/H222P mice, related to STAR Methods

Table S3. Primer listing, related to Key resources table

Document S2. Article plus supplemental information
