# Supplementary material for: The non-muscle ADF/cofilin-1 controls sarcomeric actin filament integrity and force production in striated muscle laminopathies
Source: Cell Rep. 2021 Aug 24;36(8):109601. doi: 10.1016/j.celrep.2021.109601 (PMC8411111; doi:10.1016/j.celrep.2021.109601)
Supplement: Document S1. Figures S1–S9 and Table S1 [file mmc1.pdf]

Cell Reports, Volume *36*

Supplemental information

The non-muscle ADF/cofilin-1 controls sarcomeric actin filament integrity and force production in striated muscle laminopathies

Nicolas Vignier, Maria Chatzifrangkeskou, Luca Pinton, Hugo Wioland, Thibaut Marais, Mégane Lemaitre, Caroline Le Dour, Cécile Peccate, Déborah Cardoso, Alain Schmitt, Wei Wu, Maria-Grazia Biferi, Naïra Naouar, Coline Macquart, Maud Beuvin, Valérie Decostre, Gisèle Bonne, Guillaume Romet-Lemonne, Howard J. Worman, Francesco Saverio Tedesco, Antoine Jégou, and Antoine Muchir

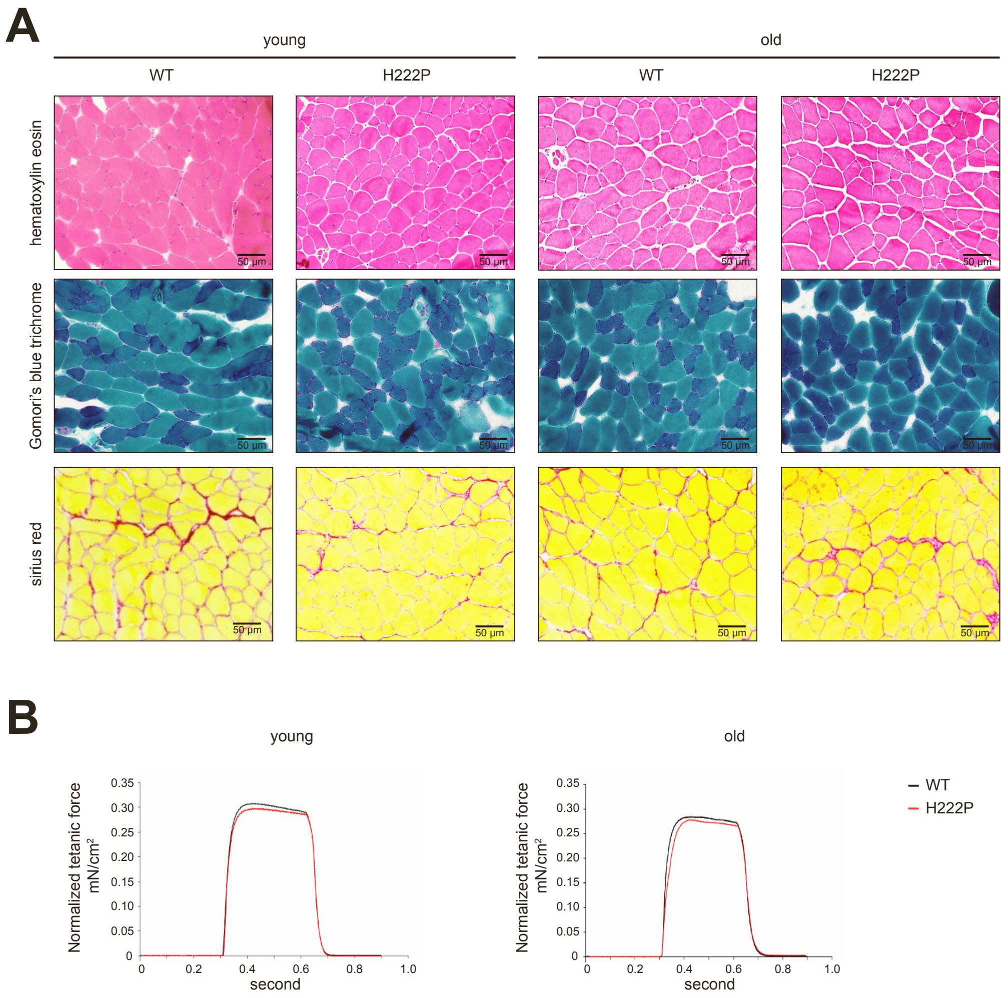

Figure S1. Skeletal muscle structure and function of fast twitch EDL muscle from *Lmna*$^{p.H222P/H222P}$ mice, related to Figure 1.

(A) Histochemical analysis of EDL from young and old, wild type (WT) and *Lmna*$^{p.H222P/H222P}$ (H222P) mice. Sections of EDL muscles were stained with hematoxylin and eosin, modified Gomori's Trichrome, and Sirius red. Scale bar = 50 µm.

(B) Representative curves of tetanic forces of EDL muscle from young and old, wild type (WT) and *Lmna*$^{p.H222P/H222P}$ (H222P) mice.

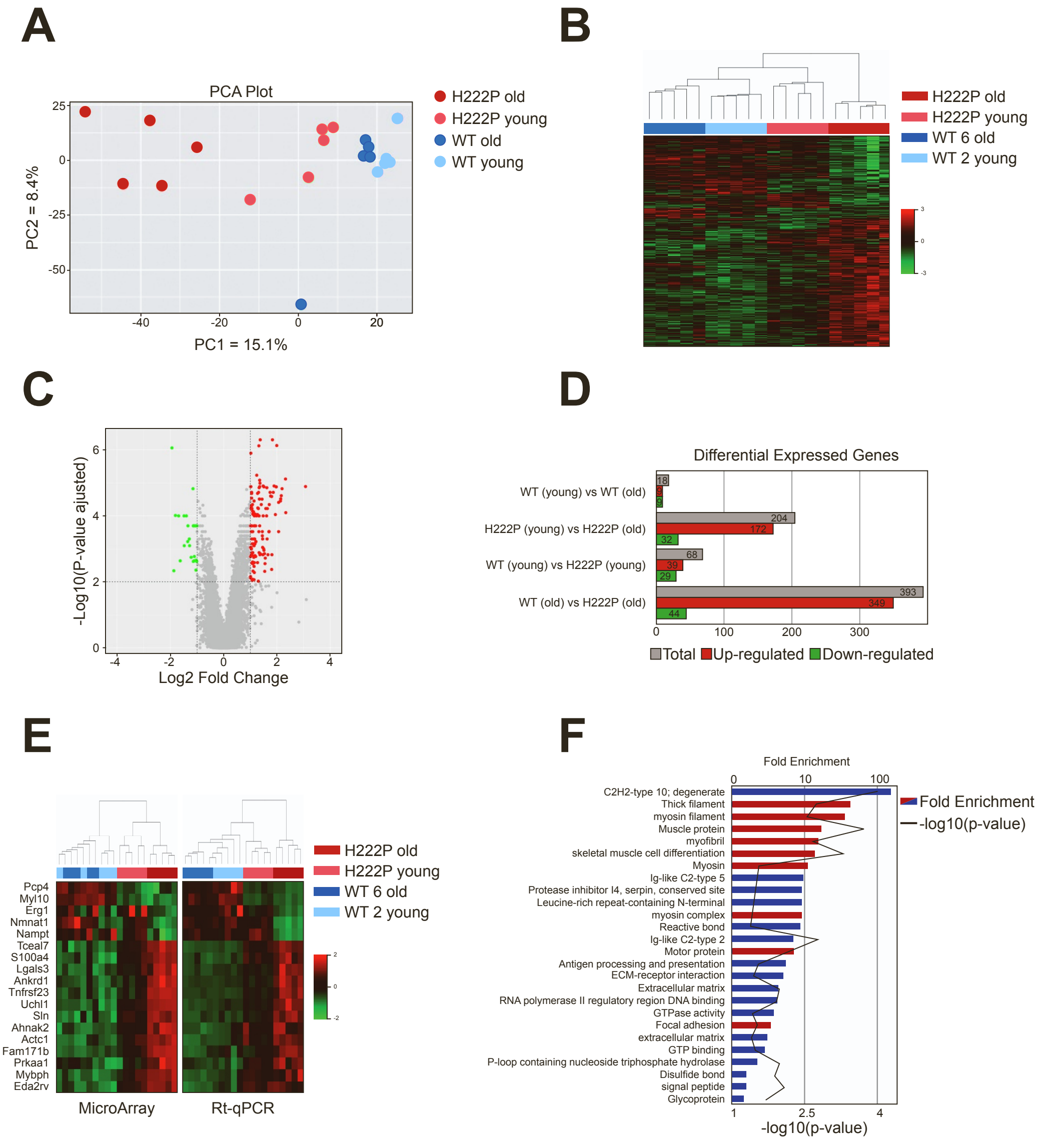

Figure S2. Transcriptomic analysis of *Lmna*$^{p.H222P/H222P}$ mice soleus muscle, related to STAR Methods.

(A) Principal component analysis (PCA) of the Affymetrix's probe sets detected in the soleus from young and old wild, type (WT) (n=5) and *Lmna*$^{p.H222P/H222P}$ (H222P) (n=5) mice.

(B) Unsupervised hierarchical clustering of the Affymetrix probe sets detected in the soleus from young and old, wild type (WT) (n=5) and *Lmna*$^{p.H222P/H222P}$ (H222P) (n=5) mice. Branch lengths of the dendrogram (top of the panel) represent similarity of the expression pattern between the samples.

(C) Volcano plot of fold change value (log2 Fold Change) and the p value (-log10 p-values adjusted) for each probe from young (n=5) and old (n=5) *Lmna*$^{p.H222P/H222P}$ (H222P) mice. A 2-fold threshold and p<0.01 were used to determine the probe set significantly altered.

(D) Schematic representation of the dysregulated genes number in soleus from young and old, wild type (WT) (n=5) and *Lmna*$^{p.H222P/H222P}$ (H222P) (n=5) mice.

(E) Supervised hierarchical clustering analysis of a set of genes quantified by Affymetrix Microarray (left panel) and RT-qPCR (right panel) in the soleus from young and old, wild type (WT) (n=5) and *Lmna*$^{p.H222P/H222P}$ (H222P) (n=5) mice.

(F) Schematic representation of the best 26 gene ontology (GO) terms score obtained from DAVID analysis of soleus from young and old *Lmna*$^{p.H222P/H222P}$ (H222P) (n=5) mice.

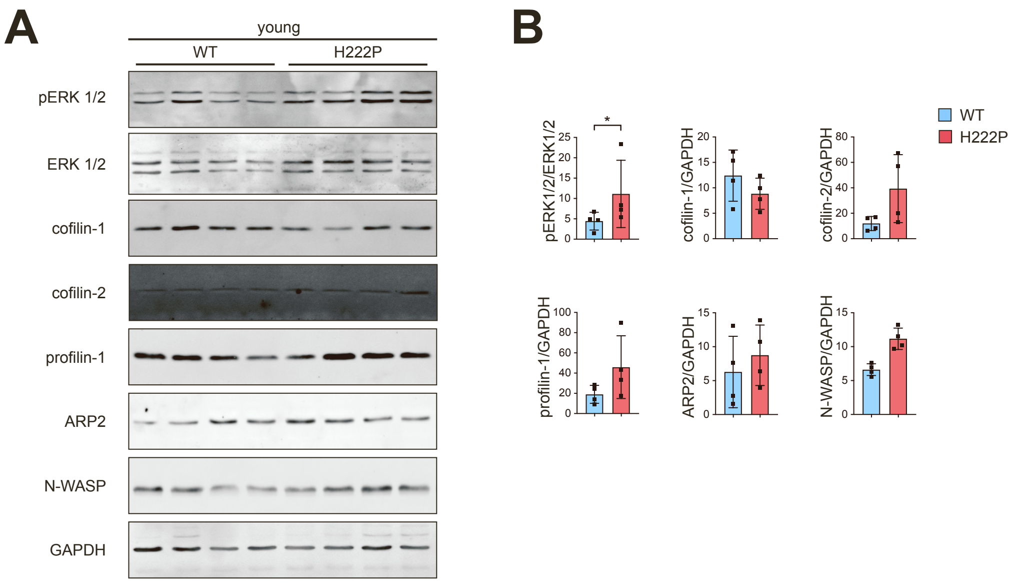

Figure S3. Cofilin-1 expression in soleus from young *Lmna*$^{p.H222P/H222P}$ mice, related to Figure 2.
(A) Immunoblots showing pERK1/2, ERK1/2, cofilin-1, cofilin-2, profilin-1, ARP2/3 and N-WASP expression levels in soleus from young wild type (WT) (n=4) and *Lmna*$^{p.H222P/H222P}$ (H222P) (n=4) mice. GAPDH is shown as loading control.
(B) Relative expression of pERK1/2, cofilin-1 and profilin-1 in the soleus from young wild type (WT) (n=4) and *Lmna*$^{p.H222P/H222P}$ (H222P) (n=4) mice. *p≤0.01 between young wild type (WT) and *Lmna*$^{p.H222P/H222P}$ (H222P). Data are represented as mean ± SD.

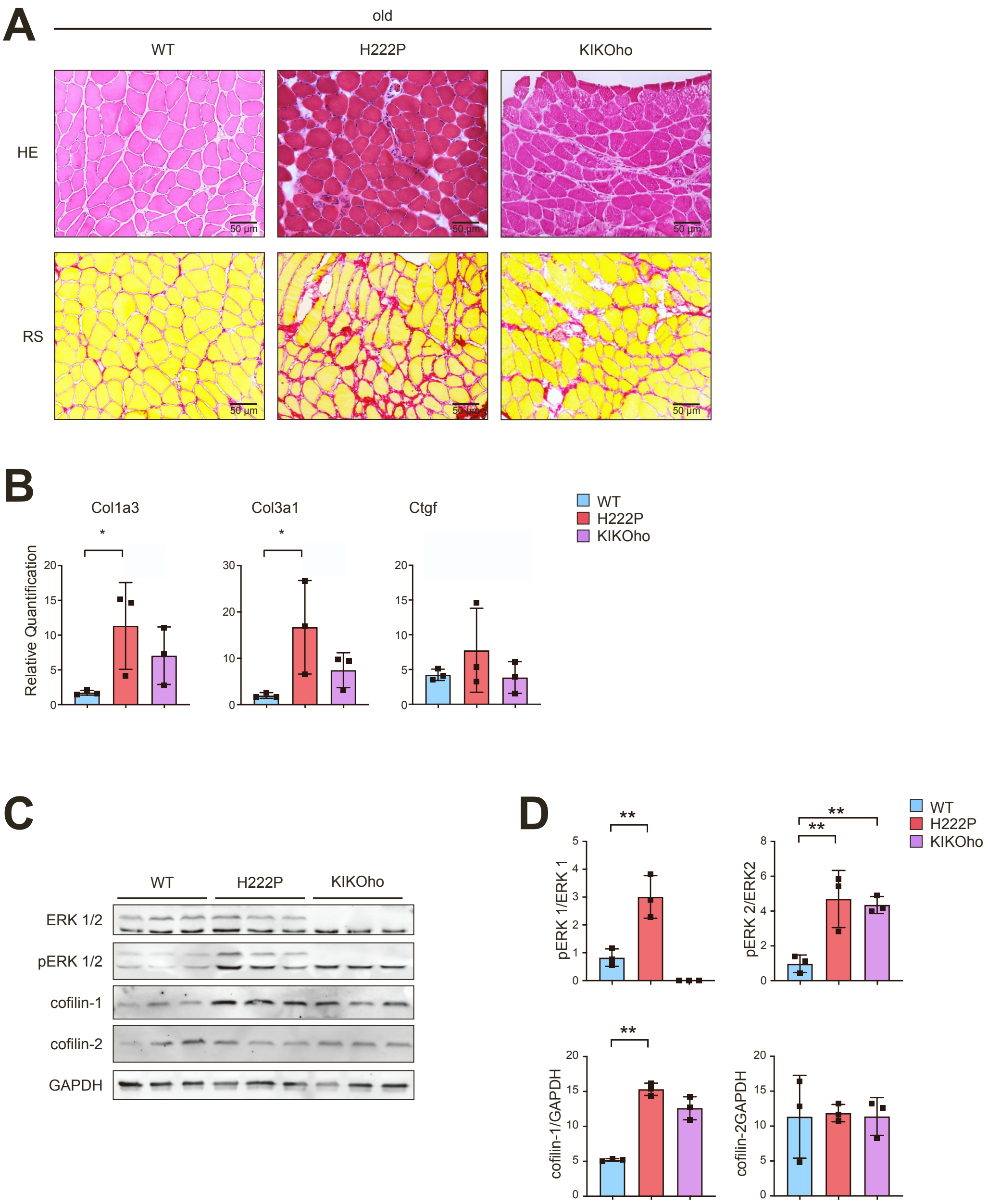

Figure S4. Loss of ERK1 in *Lmna*$^{p.H222P/H222P}$ mice improves soleus muscle integrity, related to Figure 3.

(A) Histochemical analysis of soleus from old, wild type (WT), *Lmna*$^{p.H222P/H222P}$ (H222P) and *Lmna*$^{p.H222P/H222P}$ mice lacking Erk1 (KIKOho) mice. Sections of soleus muscles were stained with hematoxylin and eosin and Sirius red. Scale bar = 50 µm.

(B) Expression of fibrosis-related genes (*Col1a2*, *Col3a1* and *Ctgf*) in the soleus from old, wild type (WT) (n=3), *Lmna*$^{p.H222P/H222P}$ (H222P) (n=3) and *Lmna*$^{p.H222P/H222P}$ mice lacking Erk1 (KIKOho) (n=3) mice. Data are represented as mean ± SD.

(C) Immunoblots showing pERK1/2, ERK1/2, cofilin-1 and cofilin-2 protein level in soleus from, old wild type (WT) (n=3), *Lmna*$^{p.H222P/H222P}$ (H222P) (n=3) and *Lmna*$^{p.H222P/H222P}$ mice lacking Erk1 (KIKOho) (n=3) mice. GAPDH is shown as loading control.

(D) Quantification of pERK1/2, ERK1/2, cofilin-1 and cofilin-2 protein level in soleus from old, wild type (WT) (n=3), *Lmna*$^{p.H222P/H222P}$ (H222P) (n=3) and *Lmna*$^{p.H222P/H222P}$ mice lacking Erk1 (KIKOho) (n=3) mice. **$p \leq 0.001$ between old, wild type (WT) and *Lmna*$^{p.H222P/H222P}$ (H222P) or *Lmna*$^{p.H222P/H222P}$ mice lacking Erk1 (KIKOho). Data are represented as mean ± SD.

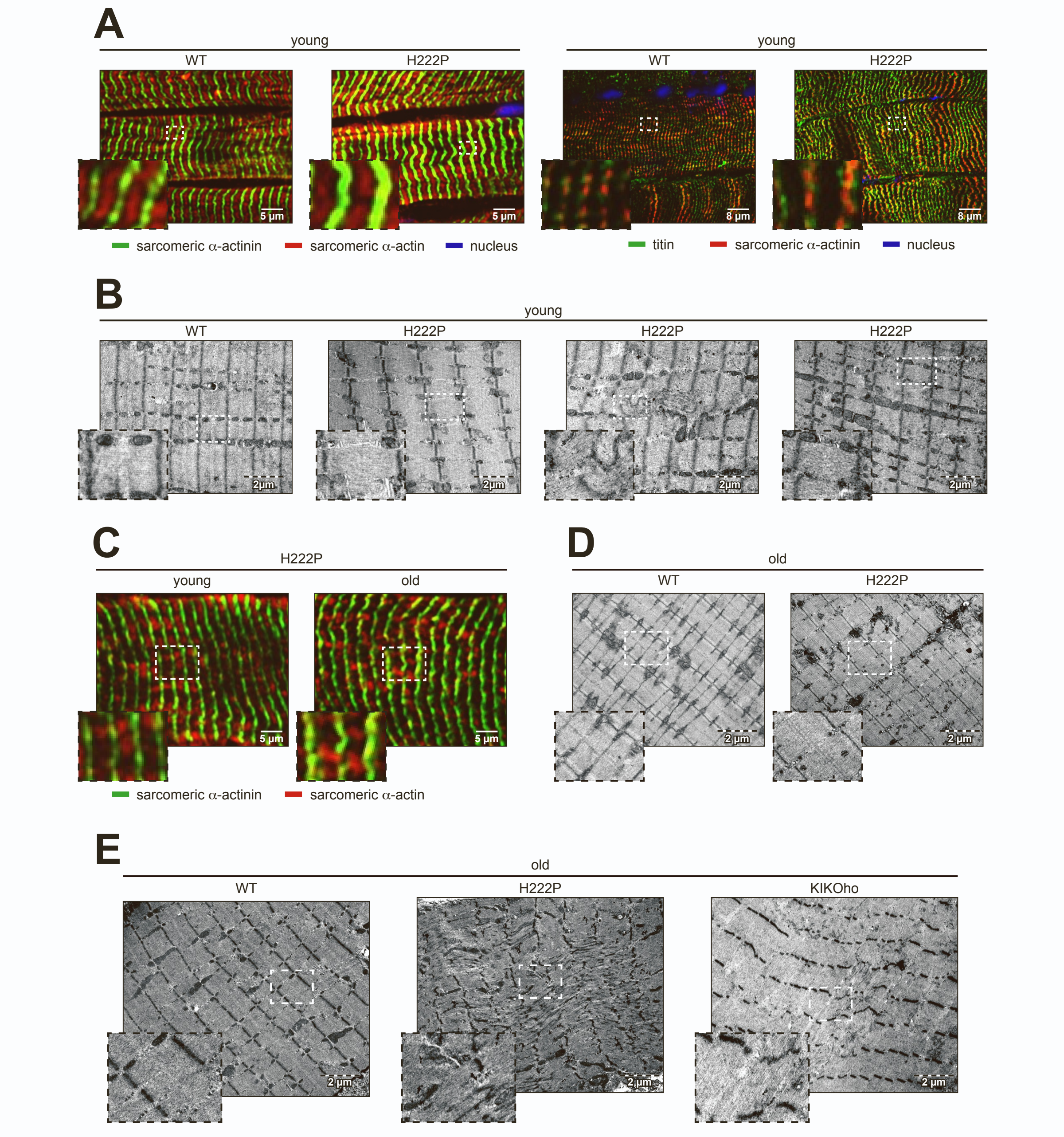

Figure S5. Sarcomeric organization of skeletal muscles from *Lmna*$^{p.H222P/H222P}$ and *Lmna*$^{p.H222P/H222P}$ mice lacking Erk1, related to Figure 5.

(A) Left: Immunofluorescence micrographs of sarcomeric α-actinin (green) and sarcomeric α-actin (red) labeled soleus muscle from young, wild type (WT) and *Lmna*$^{p.H222P/H222P}$ (H222P) mice. Sclae bar = 5 µm. Right: Immunofluorescence micrographs of titin (green) and sarcomeric α-actinin (red) labeled soleus muscle from young, wild type (WT) and *Lmna*$^{p.H222P/H222P}$ (H222P) mice. Scale bar = 8 µm

(B) Electron microscopy showing sarcomeric organization in soleus muscles from young, wild type (WT) and *Lmna*$^{p.H222P/H222P}$ (H222P) mice. Scale bar = 2 µm.

(C) Immunofluorescence micrographs of sarcomeric α-actinin (green) and sarcomeric α-actin (red) labeled EDL muscle from young and old *Lmna*$^{p.H222P/H222P}$ (H222P) mice. Scale bar = 5 µm.

(D) Electron microscopy showing sarcomeric organization in EDL muscles from old, wild type (WT) and *Lmna*$^{p.H222P/H222P}$ (H222P) mice. Scale bar = 2 µm.

(E) Electron microscopy showing improvement of sarcomeric organization in soleus muscles from old, wild type (WT), *Lmna*$^{p.H222P/H222P}$ (H222P) mice and *Lmna*$^{p.H222P/H222P}$ mice lacking Erk1 (KIKOho). Scale bar = 2 µm.

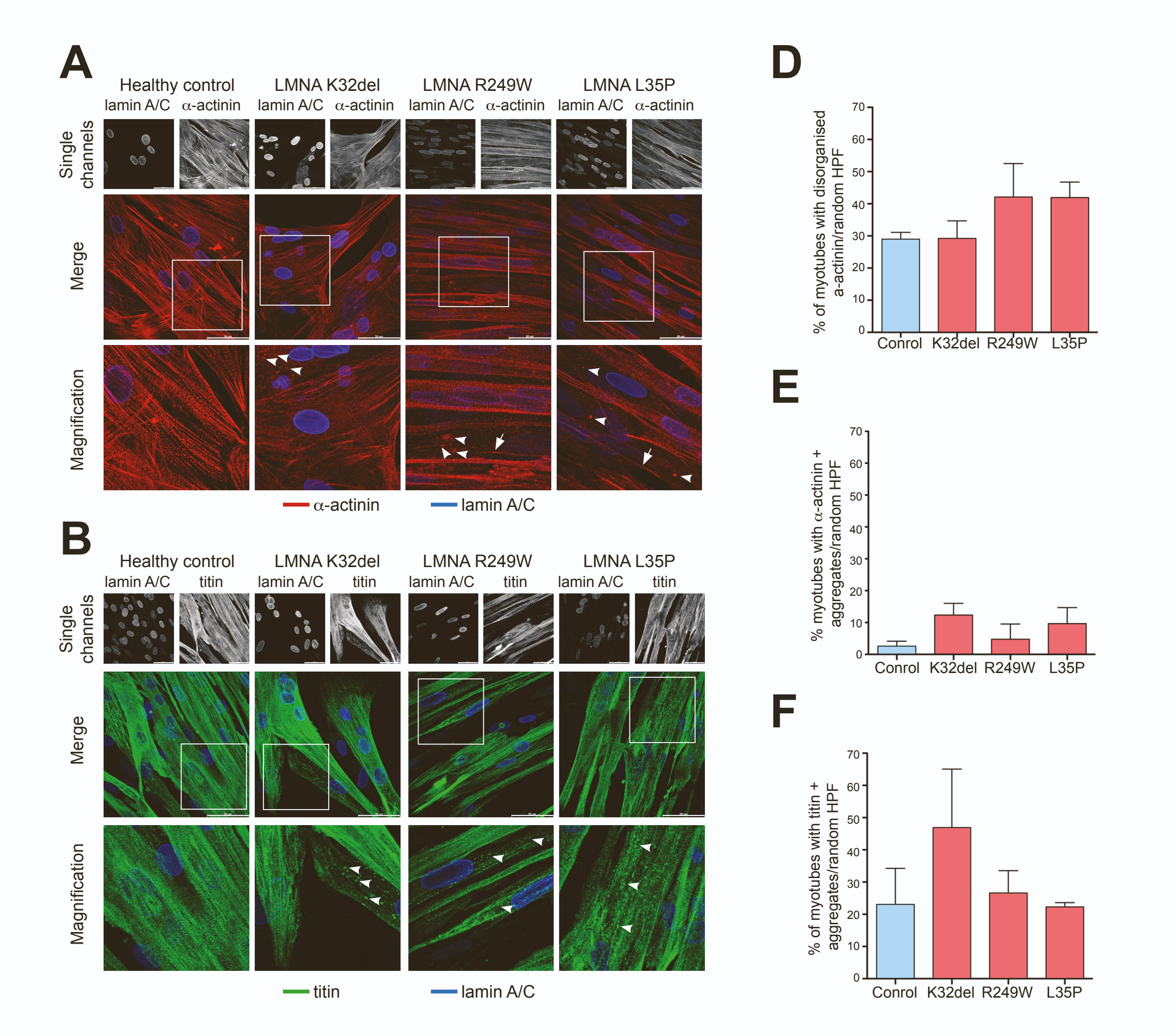

Figure S6. Analysis of sarcomeric proteins in K32del, p.R249W and p.L35P *LMNA* mutant iPSC-derived myotubes, related to Figure 5.

(A) Immunofluorescence images showing A-type lamins (blue) and sarcomeric α-actinin (red) staining of three *LMNA* mutants and one healthy control lines of human iPSC-derived myotubes. Arrows indicate myotubes with disorganised sarcomeric structure and arrowheads point at sarcomeric α-actinin aggregates. White boxes highlight magnified area in lower panels. Scale bar = 50 μm.

(B) Immunofluorescence of the three lines described in (A) showing A-type lamins (blue) and titin (green). Arrowheads indicate titin aggregates. White boxes highlight magnified area in lower panels. Scale bar = 50 μm.

(C) Graph quantifies the percentage of myotubes with disorganised/immature sarcomeres assessed by sarcomeric α-actinin staining. 9 to 15 randomly selected high-power fields (HPF) with 15 to 93 myotubes were imaged and analysed per cell line per repeat (n=3; Kruskal-Wallis test: $p = 0.1659$). Data are represented as mean ± SD.

(D) Graph quantifies the percentage of myotubes with sarcomeric α-actinin-positive aggregates. Same pictures of panel (B) were analysed. (n=3; Kruskal-Wallis test: $p = 0.2124$). Data are represented as mean ± SD.

(E) Graph quantifies the percentage of myotubes with titin-positive aggregates (n=3; Kruskal-Wallis test: $p = 0.7273$). Data are represented as mean ± SD.

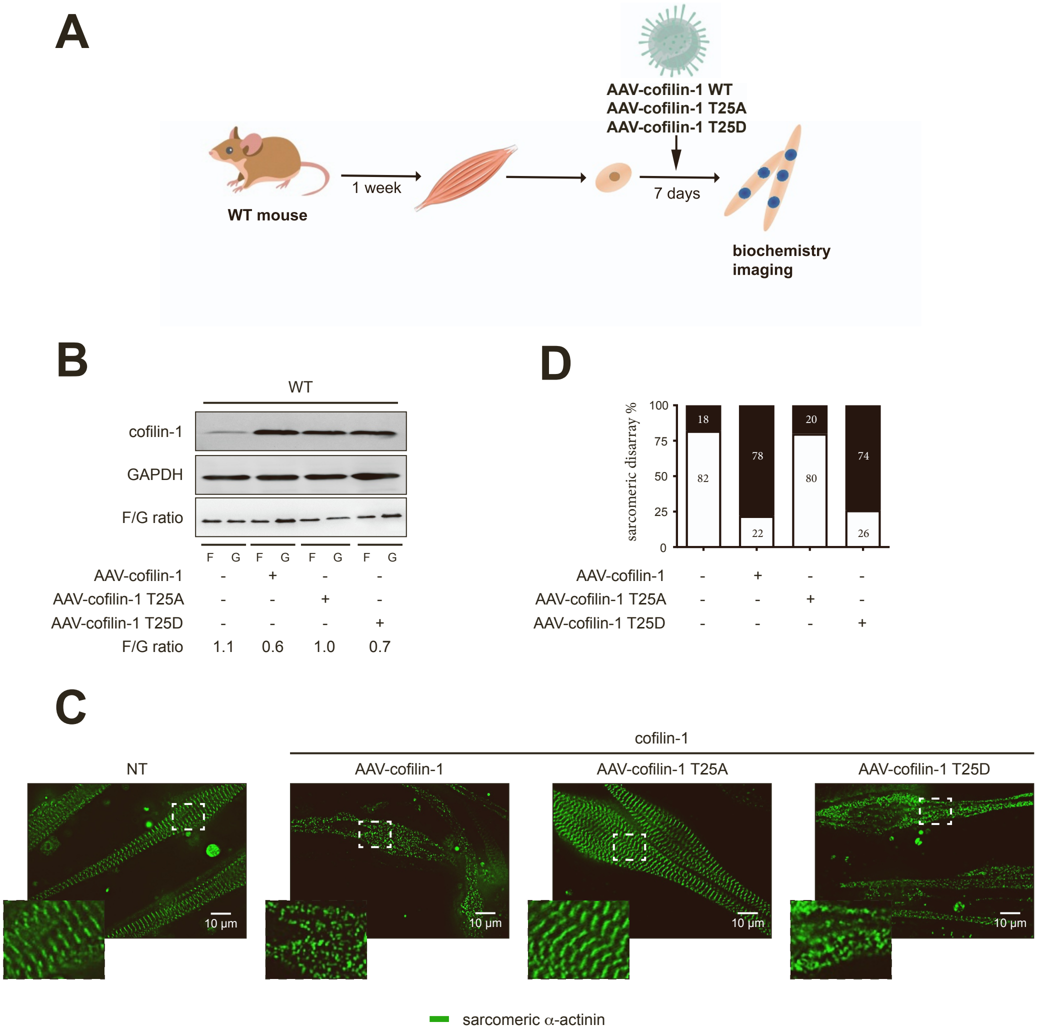

Figure S7. Phospho(T25)-cofilin-1 drives sarcomeric disorganization, related to Figure 5.

(A) Schematic representation of the experimental procedure for primary myoblasts isolation and differentiation to myotubes from 1-week-old wild type (WT) mice transduced with AAV vectors expressing cofilin-1 constructs.

(B) Immunoblots showing cofilin-1 protein expression level in myotubes derived from wild type mouse myoblasts (WT) and wild type mouse myoblast transduced with AAV vectors expressing cofilin-1 constructs. Representative immunoblot showing the effect of AAV expressing cofilin-1 constructs on G-actin and F-actin expression was shown.

(C) Immunofluorescence micrographs of α-actinin labeled myotubes (green) derived from wild type mouse myoblasts (WT) and wild type mouse myoblast transduced with AAV vectors expressing cofilin-1 constructs. Scale bar = 10 μm.

(D) Quantification of sarcomeric disarray from α-actinin labeled myotubes derived from wild type mouse myoblasts (WT) and wild type mouse myoblast transduced with AAV vectors expressing cofilin-1 constructs. (white bar: normal sarcomeric organization; black bar: sarcomeric disarray).

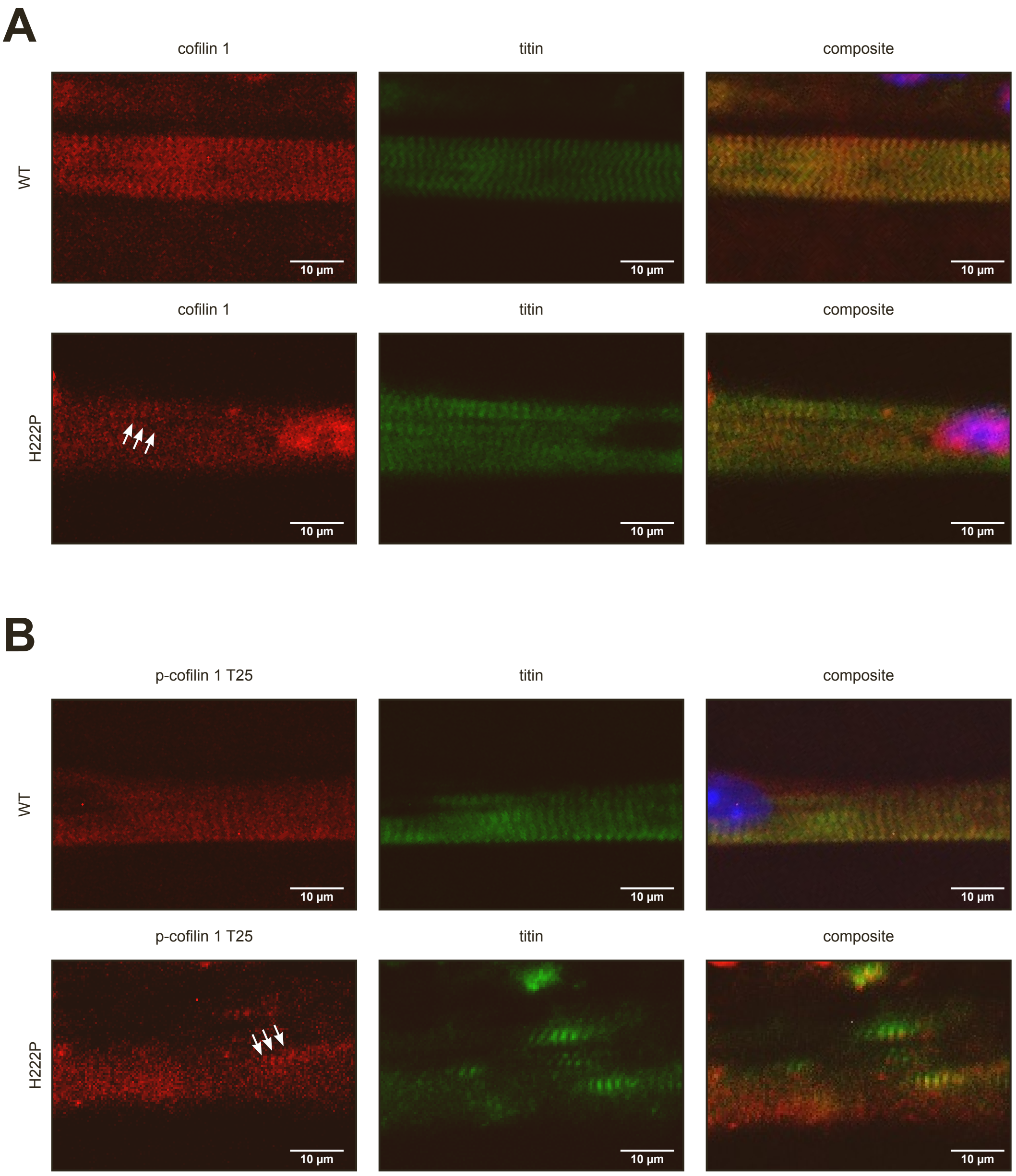

Figure S8. Localization of cofilin-1 and phospho(T25)-cofilin-1 in muscle cells, related to Figure 5.

(A) Immunofluorescence micrographs from old wild type (WT) and *Lmna*$^{p.H222P/H222P}$ (H222P) mice. Titin (green) shows the sarcomeric I-band and cofilin-1 (red) displays punctuate cytoplasmic localization (arrows), which does not overlap with myofibrils. Scale bar = 10 µm.

(B) Immunofluorescence micrographs from old wild type (WT) and *Lmna*$^{p.H222P/H222P}$ (H222P) mice. Titin (green) shows the sarcomeric I-band and phospho(T25)-cofilin-1displays punctuate cytoplasmic localization (arrows), which does not overlap with myofibrils. Scale bar = 10 µm.

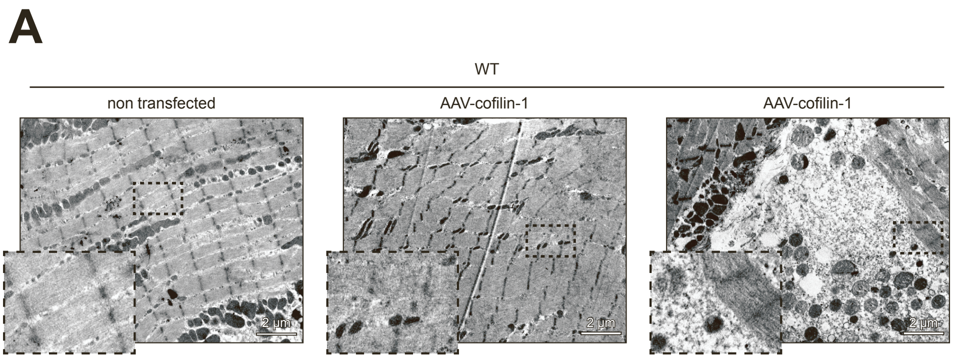

Figure S9. Cofilin-1 is involved in the sarcomere disorganization *in vivo*, related to Figure 6. Electron micrographs showing myofilaments organization in soleus from wild type (WT) mice non-transfected or transfected with AAV vector expressing cofilin-1. Scale bar = 2 µm.

	Tetanic contraction			
	Young		Old	
	WT	H222P	WT	H222P
n muscles (n mice)	12 (6)	12 (6)	14 (7)	9 (6)
BW (g)	26.7 ± 1.0	23.7 ± 1.3	27.6 ± 1.3	26.5 ± 1.9
MW (mg)	7.1 ± 0.3	5.9 ± 0.3	7 ± 0.4	6.5 ± 0.3
l_0 (mm)	10.5 ± 0.2	10.3 ± 0.3	9.8 ± 0.1	9.5 ± 0.1
P_0 (mN)	123.0 ± 8.0	109.1 ± 10.5	135.9 ± 8.7	91.4 ± 7.1**
sP_0 (mN/mm^2)	132.8 ± 9.7	136.9 ± 11.9	137.9 ± 7.0	96.3 ± 5.7***

	Twitch contraction			
	Young		Old	
	WT	H222P	WT	H222P
n muscles (n mice)	12 (6)	12 (6)	10 (5)	3 (3)
BW (g)	26.7 ± 1.0	23.7 ± 1.3	29.2 ± 1.2	26.4 ± 4.1
MW (mg)	7.1 ± 0.3	5.9 ± 0.3	7.7 ± 0.3	7.3 ± 0.7
l_0 (mm)	10.5 ± 0.2	10.3 ± 0.3	9.9 ± 0.1	9.2 ± 0.2
P_t (mN)	28.1 ± 0.4	23.9 ± 7.7	26.7 ± 0.3	17.5 ± 2.7*
sP_t(mN/mm^2)	30.5 ± 3.5	30.0 ± 2.5	25.4 ± 2.8	15.6 ± 2.1*
HRT (ms)	67.0 ± 4.3	67.3 ± 3.7	55.5 ± 5.3	57.7 ± 9.0
TTP (ms)	43.3 ± 2.0	46.2 ± 1.7	44.1 ± 2.5	41.7 ± 4.8

Table S1. Ex vivo soleus tetanic and twitch force measurements from young and old, wild type (WT) and *Lmna*$^{p.H222P/H222P}$ (H222P) mice, related to Figure 1.

Data are represented as mean ± SD. *p≤0.01, **p≤0.001, ***p≤0.0001 between old, wild type (WT) and *Lmna*$^{p.H222P/H222P}$ (H222P).
